# Heart Substructure Radiation Dose and Cardiac Outcomes: Contemporary Evidence and Actionable Opportunities: *JACC CardioOncology* State-of-the-Art Review

**DOI:** 10.1016/j.jaccao.2026.04.009

**Published:** 2026-06-16

**Authors:** Katelyn M. Atkins, Gerard Walls, Andriana P. Nikolova, Joel Ferrall, Maria Oorloff, Jakub Bychowski, Kollin Kolb, Anja Karlstaedt, Joshua D. Mitchell, Raymond H. Mak, Carmen Bergom

**Affiliations:** a Department of Radiation Oncology, Samuel Oschin Cancer Center, Cedars-Sinai Medical Center, Los Angeles, California, USA; b Department of Cardiology, Smidt Heart Institute, Cedars-Sinai Medical Center, Los Angeles, California, USA; c Johnston Cancer Research Centre, Queen's University Belfast, Belfast, Northern Ireland, United Kingdom; d Cancer Centre Belfast City Hospital, Belfast Health & Social Care Trust, Belfast, Northern Ireland, United Kingdom; e Department of Radiation Oncology, Cardio-Oncology Center of Excellence, Alvin J. Siteman Center, Washington University, St Louis, Missouri, USA; f 1st Department of Cardiology, Medical University of Gdansk, Gdansk, Poland; g Department of Medicine, Cardio-Oncology Center of Excellence, Alvin J. Siteman Center, Washington University, St Louis, Missouri, USA; h Department of Radiation Oncology, Brigham and Women's Hospital and Harvard Medical School, Boston, Massachusetts, USA

**Keywords:** cardiac events, cardiac substructure, cardiac toxicity, LAD, MACE, radiation therapy

## Abstract

Contemporary cardiac outcomes research demonstrates that radiation dose to discrete cardiac substructures, particularly the coronary arteries, left ventricle, and pulmonary veins, strongly predict ischemia, heart failure, and arrhythmia endpoints, respectively, compared to whole heart metrics. This rapidly expanding literature has produced an increasingly rich but heterogeneous evidence base, underscoring both substantial progress and a persistent gap in clinical implementation. Importantly, current cardio-oncology guidelines and expert panel statements offer comprehensive cardiovascular recommendations, but integration of cardiac radiation dose exposure thresholds that translate into actionable clinical guidance remains limited. This partial alignment between emerging evidence and formal guidance contributes to uncertainty in clinical adoption and is further compounded by workflow complexity, limited consensus, and sparse clinical trial integration. In this State-of-the-Art Review, we synthesize contemporary cardiac dosimetry evidence on cardiac outcomes, propose a pragmatic, clinically-oriented framework, and outline actionable strategies to shorten the evidence-to-practice gap in the modern radiotherapy care continuum.

## Highlights

• Cardiac radiation exposure is linked with increased risk of cardiac events.
• Modern studies link cardiac substructure radiation dose to specific cardiac endpoints.
• Prioritizing left coronary artery dose-sparing is evidence-informed and clinically feasible.
• Pragmatic, evidence-based cardiac dose limits can narrow the evidence-to-practice gap.

Over the past decade, our understanding of radiation-associated cardiac dysfunction has advanced from a whole-heart dosimetry and overall survival-based perspective to a refined, mechanistic era grounded in cardiac substructure-specific biology and modern cardiac outcomes research.^1^, ^2^, ^3^, ^4^ Early work established that cardiac dose exposure matters,^5^ and that clinically meaningful cardiovascular events occur earlier and more frequently than once appreciated,^6^, ^7^, ^8^ challenging long-held assumptions about latency. The next wave of literature revealed that discrete cardiac substructures, particularly the coronary arteries,^9^, ^10^, ^11^, ^12^, ^13^ left ventricle,^9^^,^^14^ and pulmonary veins (PVs),^15^, ^16^, ^17^ frequently outperform whole-heart dose metrics in predicting ischemia, heart failure (HF), and arrhythmia endpoints, respectively. Recently, the rapid expansion of observational, imaging, biomarker, and modeling studies has produced an increasingly rich but heterogeneous evidence base,^1^, ^2^, ^3^^,^^18^ highlighting both substantial progress and a persistent clinical implementation gap. Current guidelines reflect this tension: the National Comprehensive Cancer Network disease-site guidelines provide limited cardiovascular risk guidance and primarily whole-heart dose metrics (with Hodgkin lymphoma as a notable exception with inclusion of comprehensive substructure dose limits), whereas cardio-oncology society statements offer comprehensive cardiovascular care recommendations but with only limited guidance on cardiac radiation dose exposure thresholds. This partial alignment between emerging evidence and formal guidance can create uncertainty about clinical adoption. Further translation has been hindered by cardiac substructure contouring and workflow complexity, a seemingly overwhelming number of potential substructures and dose constraints, and a persistent lack of cardiac radiation information in the clinical record available to treating physicians, in addition to the limited guidelines.

To bridge this gap, a practical, clinically-oriented framework is necessary; one that integrates three foundational pillars of cardiovascular risk estimation across the treatment timeline: 1) baseline cardiovascular risk assessment, 2) high-value, selective cardiac substructure dosimetry, and 3) actionable strategies for clinical risk reduction that can be realistically implemented in routine radiation oncology practice (Central Illustration).

**Central Illustration:**
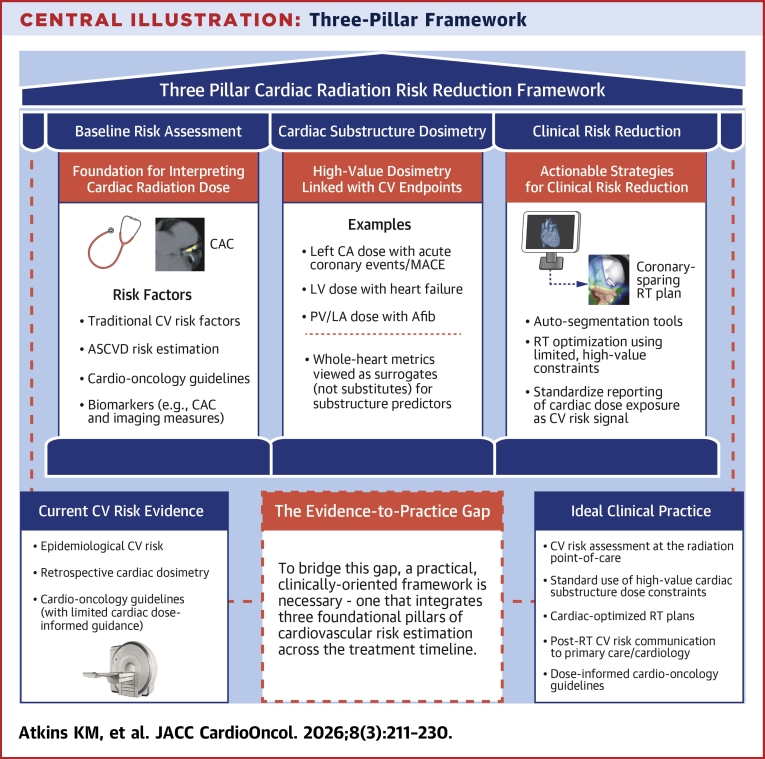
Three-Pillar Framework A practical three-pillar framework for clinical cardiovascular risk reduction related to cardiac substructure radiation exposure. Afib = atrial fibrillation; ASCVD = atherosclerotic cardiovascular disease; CA = coronary artery; CAC = coronary artery calcium; CV = cardiovascular; LA = left atrium; LV = left ventricle; MACE = major adverse cardiac event; PV = pulmonary valve; RT= radiotherapy.

In this State-of-the-Art Review, we synthesize contemporary evidence on heart dosimetry and cardiac outcomes, articulate a practical three-pillar framework, and outline concrete, actionable pathways to shorten the evidence-to-practice gap and reduce radiation-associated cardiac risk in the modern cardio-oncology era.

## Pillar 1

### Baseline cardiovascular risk: the foundation for interpreting cardiac radiation dose

Highlights:

• Baseline cardiovascular health informs treatment decisions, supports targeted cardio-oncology referrals, and underpins accurate interpretation of radiation-associated cardiac risk.
• Standardized cardiovascular risk factors should be collected in all patients to permit atherosclerotic cardiovascular disease (ASCVD) risk estimation.
• Coronary artery calcium (CAC) on radiotherapy planning computed tomography (CT) scans is a high-value, opportunistic marker of atherosclerotic burden at the radiation oncology point of care that can re-classify risk and predict cardiac events independent of dose.

#### Why baseline cardiovascular risk matters in radiation-associated heart dysfunction

Baseline cardiovascular risk substantially influences both the absolute and, in selected contexts, the relative hazard risk conferred by cardiac radiation dose.^19^ Patients with cancer often harbor elevated cardiovascular risk at cancer diagnosis, driven by traditional risk factors, systemic inflammation, accelerated vascular aging, and medical under-optimization.^20^ As a result, baseline cardiovascular health is essential for contextualizing radiation dose-response relationships across cardiac dosimetry and disease-site literature.

#### Traditional cardiovascular risk models are necessary but insufficient

In the United States, the most widely used tool for estimating 10-year ASCVD risk and guiding statin therapy per American College of Cardiology (ACC)/American Heart Association (AHA) guidelines is the Predicting Risk of Cardiovascular EVENTs (PREVENT) equations.^21^ However, these models do not incorporate cancer exposures as risk factors, therefore potentially systematically underestimating cardiovascular risk.^20^ Studies have consistently demonstrated that patients with cancer are not medically optimized, with substantial rates of cardiac disease.^22^ Furthermore, a growing body of evidence supports the cardioprotective role of statin therapy in patients treated with thoracic or head/neck radiotherapy, beyond traditional risk score stratification.^23^^,^^24^

#### Radiographic cardiovascular risk markers embedded in routine radiotherapy workflows

Coronary artery calcium (CAC) is a powerful marker of atherosclerotic burden and identifies patients who are at known increased risk of cardiovascular events, and thus may benefit from statin therapy initiation or dose intensification.^25^ Several studies have demonstrated CAC can be readily identified and accurately quantified on radiotherapy planning CT scans.^26^^,^^27^ Furthermore, CAC measured from radiotherapy planning CT scans has been linked with cardiac events and mortality in lung cancer^10^^,^^27^^,^^28^ and acute coronary events and cardiovascular disease in breast cancer.^26^^,^^29^ Nearly half of asymptomatic breast cancer survivors in the prospective CAROLE (Cardiac-Related Oncology Late Effects) study were found to have clinical coronary artery disease (CAD) on CAC screening,^30^ identifying a readily actionable risk modifier in the cancer treatment continuum. Lastly, if oncologists observe the presence of CAC during routine care, this finding should be communicated to patients and primary care physicians for clinical follow-up.

Emerging data have identified other pre-treatment CT-derived heart parameters as associated with cardiac outcomes and mortality.^14^^,^^31^^,^^32^ Baseline right and left volume indices (LAVI) have been associated with major adverse cardiac events (MACE) after chest radiotherapy,^31^ including associations of LAVI with heart failure with preserved ejection fraction (HFpEF), left atrium (LA) volume with atrial arrhythmia, and left ventricle (LV)/right ventricle (RV) volume ratio with HF.^32^ Furthermore, epicardial fat volume is a marker of cardiometabolic vulnerability and has been associated with the risk of MACE after thoracic RT.^33^ Taken together, these studies support continued investigation into optimizing personalized cardiovascular risk assessment at the radiation oncology point-of-care.

## Pillar 2

### High-value cardiac substructure dosimetry linked with clinical cardiovascular endpoints

Highlights:

• Meaningful interpretation of cardiac dosimetry requires linking substructure radiation dose to mechanistically plausible cardiovascular endpoints, such as.
  ○ Left coronary (left main coronary artery [LMCA], left anterior descending coronary artery [LAD], left circumflex [LCx]) dose with acute coronary events/MACE.
  ○ LV myocardium dose with subclinical dysfunction, HF.
  ○ PVs/LA dose with atrial fibrillation.
  ○ Cardiac valve dose with valvular disease.
• Composite cardiac endpoints and non-anatomic regions of interest can obscure dose-response relationships, particularly when event composition or baseline cardiovascular risk varies across cohorts.
• Whole-heart dose metrics (eg, mean heart dose [MHD]) should be viewed as surrogates (not substitutes) for mechanistic, endpoint-aligned dosimetric predictors.

#### Rationale for cardiac substructure dosimetry

**Figure 1:**
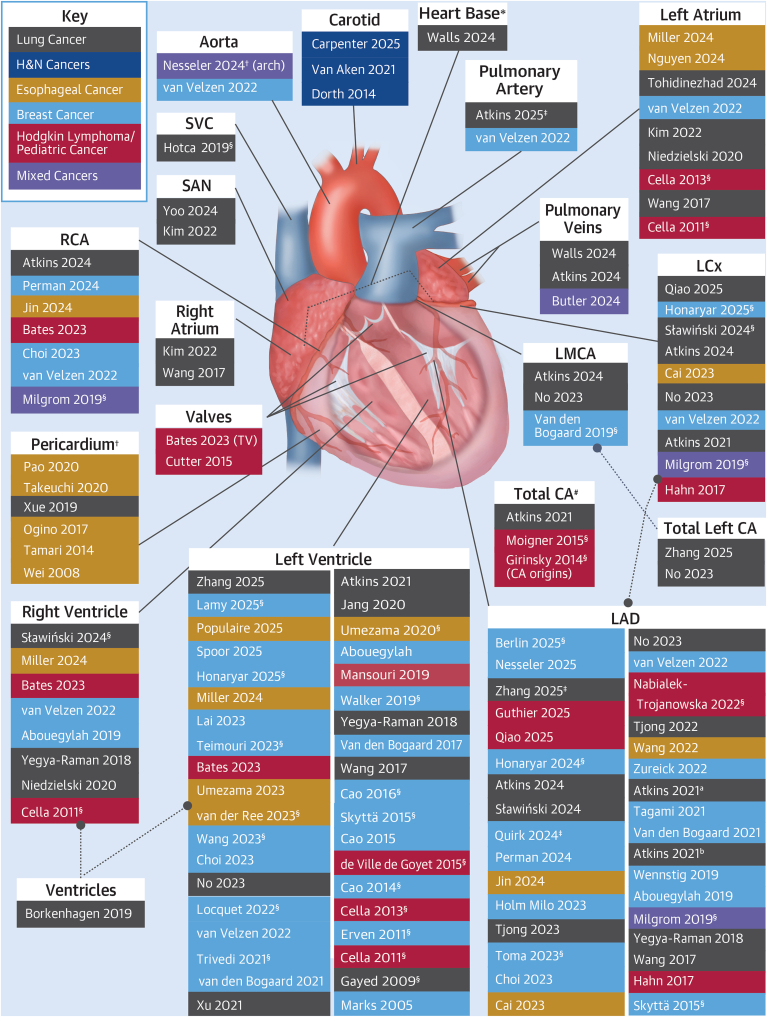
Studies Linking Cardiac Substructure Radiation Exposure With Cardiac-Specific Outcomes Studies denoted by first author and year of publication. Studies describing associations between whole heart doses and outcomes, or sub-structures and survival only, were excluded. Studies where substructure dose predictors were not statistically significant on multi-variable analysis were excluded from illustration but included in the Supplemental Table. ∗Heart base per Walls et al. 2024 includes the RA, SVC, aortic root, LMCA, proximal LAD, and RCA. ^†^Pericardium, not including heart. ^ˆ^Carotid, including carotid bulb. ^‡^Study is published in pre-print and/or abstract (≥2024) only. ^#^Composite of all coronary structures (except where noted as CA origins). ^§^Study with subclinical cardiovascular endpoint only. CA = coronary artery; H&N = head and neck; LAD = left anterior descending coronary artery; LCx = left circumflex coronary artery; LMCA = left main coronary artery; myo = myocardium; pulm = pulmonary; RCA = right coronary artery; SAN = sinoatrial node; SVC = superior vena cava.

**Figure 2:**
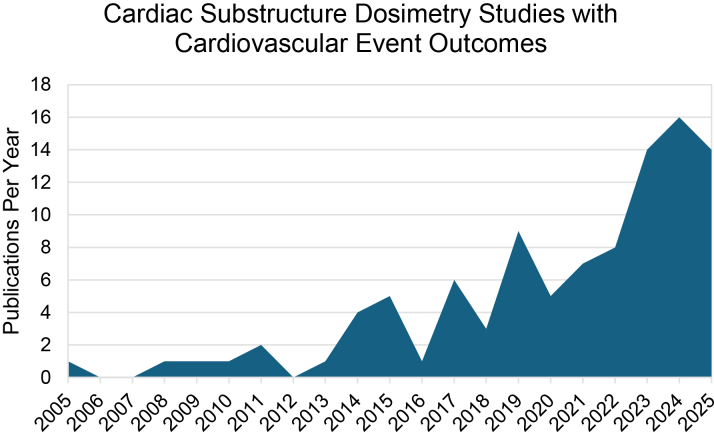
Cardiac Substructure Dosimetry Publication Trends Publication trends of cardiac substructure dosimetry studies with cardiovascular event outcomes from 2005 to 2025

Historically, cardiac dosimetry studies relied on survival endpoints, limited substructure analyses (ie, chambers, pericardium only), small sample sizes/limited events, or templated/non-personalized dosimetry for large cohorts (due to the laborious nature of data collection).^1^^,^^2^^,^^4^ Furthermore, when cardiac endpoints have been utilized, due to limited cohort sizes and event numbers, they have often relied on broad composite endpoints (such as combining arrhythmia, pericardial, ischemic, and HF outcomes),^1^^,^^3^^,^^4^ obscuring critical structure–function relationships and hindering rational selection of meaningful dosimetric predictors. Contemporary evidence, particularly in the past 5 years, now demonstrates that *specific substructure injuries map to mechanistically plausible cardiac events*, often with greater predictive strength than whole-heart metrics^9^, ^10^, ^11^, ^12^^,^^34^, ^35^, ^36^ (Supplemental Table, Figures 1 and 2). In the following section, we review the contemporary cardiac dosimetry literature to inform a substructure-endpoint-based approach for interpreting cardiac radiation exposure.

#### The importance of cardiovascular endpoint selection

##### Optimal terminology for cardiac outcomes following radiotherapy

Given the breadth of terms used in the literature, it is worth clarifying that most dose-outcomes data are based on associations, not causation, and therefore, the term “radiation-associated” is preferred over “radiation-induced.” Furthermore, to facilitate consistency in the description of risks after multi-modal therapies, we prefer the reference to cardiac “side-effects” or “events” over “toxicity,” as this is consistent with existing terminology related to systemic and other local therapies and avoids an over-interpretation of “toxicity” related to radiotherapy as a modality.

##### Mechanism-specific vs composite endpoints

Cardiac endpoints differ markedly in their pathophysiology, latency, susceptibility to baseline cardiovascular risk, and frequency of occurrence. Thus, while the blending of event types may increase statistical power, it may also dilute substructure-specific signals or alter which candidate predictors appear most informative. For instance, arrhythmias and coronary ischemic events comprise variable fractions of pooled event totals but are driven by categorically distinct substructure injury patterns. Acute coronary events are highly correlated with coronary radiation exposure,^14^^,^^37^^,^^38^ while arrhythmias (most commonly atrial fibrillation) are linked to PV exposure.^15^, ^16^, ^17^ Zhang et al. further informed this anatomic specificity by demonstrating that left ventricular myocardial dose exposure was associated with incident heart failure with reduced ejection fraction (HFrEF), while LAD and total left coronary dose exposure were not, and the latter was specifically associated with acute coronary events.^14^ Thus, a “best” or “top” dosimetric predictor in a particular dataset will depend heavily on the event distribution.

Furthermore, not all composite cardiac endpoints are created equally. For example, the ACC/AHA-defined MACE are typically reported as a five-point composite of myocardial infarction, HF, coronary revascularization, unstable angina, and cardiovascular death, with or without ischemic stroke,^39^ but these do not capture the full range of radiation-associated cardiovascular injury. Cardiac common terminology criteria for adverse events (CTCAE) classes from the National Cancer Institute more broadly capture these entities, including arrhythmia, non-ischemic HF/cardiomyopathy, myocarditis, other conduction abnormalities (e.g., QTc prolongation), pericardial, and valvular events,^40^ although they do not include sub-categorizations for HF, such as ischemic vs non-ischemic, nor separate diastolic dysfunction or HFpEF.^14^ Furthermore, event occurrence and severity grading can be impacted by the stringency of the criteria to substantiate the event. For instance, the criteria to substantiate an AHA/ACC-defined MACE of HF are more stringent than those for a “grade ≥3” HF, resulting in more than twice the HF events for grade ≥3 CTCAE vs MACE definitions (61 vs 28 events, respectively, in Atkins et al.^8^).

#### Cardiac dosimetry: coronary arteries

##### Coronary artery dosimetry in breast cancer

A dose-dependent association between whole-heart radiation exposure and ischemic heart disease was powerfully demonstrated by the landmark study by Darby et al. in 2013, which reported a linear increase in the rates of major coronary events of 7% per Gy of MHD.^5^ Subsequent studies in breast cancer survivors revealed a dose-dependent association between LAD dose exposure and high-risk plaque/stenosis,^37^^,^^41^^,^^42^ refining these observations and persisting in modern cohorts.^13^ Notably, while several large lung and esophageal cancer cardiac dosimetry studies have performed comprehensive cardiac dosimetric evaluations, including volumetric coronary dose exposure variables and testing cut-points/thresholds, breast cancer studies have been more reliant on LAD mean or maximum (max) dose alone, select continuous LAD variables (eg, volume [V] receiving 5 Gy [V5], V30, etc.), or evaluation of LV dose parameters in the absence of LAD (likely due to the technical challenges and laborious nature of LAD dose estimation).^1^^,^^36^^,^^43^^,^^44^

A 2019 study by Wennstig et al. reported that among 182 women with breast cancer treated between 1992 and 2012 who subsequently underwent coronary angiography, doses of 5 to 20 Gy or >20 Gy to the mid-LAD correlated with increased odds for coronary intervention.^37^ A 2021 study by Tagami et al. among 94 patients with breast cancer treated between 2006 and 2019 observed a 21% higher incidence of LAD CAD per Gy mean LAD dose by coronary CT angiography.^42^ These findings appear heightened in patients with pre-existing coronary plaque. In a 2021 study from van den Bogaard et al. of 910 patients with breast cancer, including 163 with baseline LAD atherosclerotic plaque, the strongest predictor of acute coronary events was mean dose to the LAD plaque.^41^ A study by Zureick et al. in 2022 reported a modern dosimetric analysis of 375 patients treated between 2012 and 2018 and identified cut-points of mean LAD ≥3 Gy and max ≥7 Gy as predictive of MACE, although they did not evaluate volumetric predictors.^13^ In this study, 6% of patients experienced a major cardiac event (HF, myocardial infarction, unstable angina, coronary revascularization, cardiac death), and 10% experienced “any” cardiac event (including arrhythmia, pericardial, valvular events).

Several studies have analyzed coronary dosimetric predictors of LV dysfunction (LVEF decline) or global longitudinal strain (GLS), though results are heterogeneous. A study by van den Bogaard et al. in 2019^44^ identified LMCA max as associated with LV GLS (but not LVEF decline), and Toma et al. in 2023^45^ observed LAD mean and max doses >33 Gy and >47 Gy, respectively, as associated with a >5% decline in LVEF in patients treated with anthracycline-based chemotherapy with or without trastuzumab-based therapy. Notably, the German Society for Radiation Oncology (DEGRO) breast cancer expert panel^46^ recommends mean LAD <10 Gy, LAD V30 and Gy <2%, and LAD V40 Gy <1% (as well as mean LV <3 Gy, LV V5 of <17%), though these LAD cut-points are not based on any explicit testing of these thresholds but are often clinically reasonable planning goals.

In echocardiography-based functional studies, there is a growing body of work demonstrating that modern radiotherapy techniques result in modest perturbations in cardiac function but without overt clinical LVEF reduction.^47^, ^48^, ^49^, ^50^ Notably, a 2025 longitudinal cohort study by Berlin et al. of 303 patients evaluated echocardiography-derived measures of cardiac function.^47^ In the setting of a median MHD and LAD dose of 2.3 Gy and 40 Gy, respectively, for left-sided radiotherapy, the max LAD dose was observed to be the strongest predictor (vs MHD) of worsening LVEF decline, strain, and diastolic function, even among all chemotherapy groups.

##### Coronary artery dosimetry in Hodgkin lymphoma and pediatric cancer

In Hodgkin lymphoma, volumetric left coronary dose exposure, including LAD V5 Gy and LCx V20 Gy (no cut-points tested) have been associated with ischemic coronary events.^51^ In a 2017 study by Hahn et al. of 125 Hodgkin lymphoma patients with reconstructed 3-dimensional treatment plans, investigators notably demonstrated that the models using coronary artery predictors outperformed those with whole-heart predictors for ischemic cardiac event endpoints but not for the composite endpoint of “any” adverse cardiac event, likely reflecting the more heterogeneous pool of events in the group. A detailed 2022 analysis by Bates et al. in 25,481 pediatric cancer survivors treated from 1970 to 1999 in the Childhood Cancer Survivor Study using reconstructed radiation doses (12,288 received radiotherapy) reported a 4% 35-year (from diagnosis) cumulative incidence of CAD and identified right coronary artery (RCA) mean 5 to 9.9 Gy as correlated with an increased risk of CAD.^35^

##### Coronary artery dosimetry in lung cancer

The extent of cardiac radiation exposure in lung and esophageal cancer treatment can often be an order of magnitude higher than that in breast radiotherapy (e.g., MHD 10-20 Gy vs 1-2 Gy), leading to increased cardiac event rates (10% to 30% overall experiencing any cardiac event) with shorter latency to onset (typical median onset within 2 years).^6^, ^7^, ^8^^,^^10^, ^11^, ^12^ The bulk of contemporary cardiac dosimetry literature has originated from landmark locally advanced lung cancer studies, refining our understanding of mechanistically plausible cardiac dosimetry.

In a 2021 large single institution retrospective analysis of 701 patients with locally advanced non–small cell lung cancer (NSCLC) treated between 1998 and 2014, Atkins et al. reported a 4% 1-year MACE cumulative incidence at a median of 21 months. They identified that the LAD V15 Gy ≥ 10% was associated with an increased risk of MACE and mortality, particularly among patients without known coronary heart disease (CHD). The risk of MACE was also correlated with the LCx V15 Gy ≥ 14% and mean total coronary artery dose ≥7 Gy. Specifically, among patients without CHD, there was an approximately 5% absolute increase in 1-year MACE rates when these thresholds were exceeded (5% vs 0%, respectively). Notably, the effect of coronary dose exposure on the risk of MACE was more pronounced in patients without overt CHD. The reasons for this observation are unclear but may relate to a greater susceptibility to radiotherapy-induced microvascular injury or myocardial fibrosis.^52^

In lung cancer, the association of LAD V15 Gy ≥ 10% with MACE was subsequently validated in an independent 2023 study by Tjong et al.,^53^ and the association with mortality was observed in a 2023 study by McKenzie et al. re-analyzing RTOG 0617^54^ and a 2024 study by Yegya-Raman et al.^55^ Notably, in the Yegya-Raman et al. study of 335 patients,^55^ no cardiac dose parameter was significantly associated with MACE, possibly owing to the high baseline cardiovascular risk (48% with known CHD) and lower overall coronary radiotherapy exposure (median LAD V15 Gy of only 1%) in the setting of a 35% rate of proton radiotherapy, suggesting that the cohort may have been underenriched for coronary dose-driven cardiovascular events.

In a 2023 study, No et al. analyzed 233 patients with locally advanced NSCLC treated between 2006 and 2018 and further validated the link between left coronary (left main, LAD, LCx) dose exposure and grade ≥3 cardiac CTCAE (predominantly conduction and myocardial events). The authors reported a 22% overall cardiac event rate at a median of 22 months and re-demonstrated the significance of V15 Gy exposure in relation to adverse cardiac events, identifying total left coronary V15 Gy ≥2.5 cc, LAD V15 Gy ≥1.5 cc, and baseline CAC as associated with adverse cardiac events.^10^ To further dissect the importance of left coronary dose exposure in predicting cardiac event types, Zhang et al. reported in a 2025 study that LAD V15 Gy ≥1 cc (the optimal absolute cut-point;^9^^,^^56^ analogous to ≥10% of the LAD) performed comparably to total left V15 Gy ≥ 2.5 cc in predicting incident acute coronary events, suggesting limited additional explanatory power with a total left coronary structure (versus an isolated LAD structure) in this context.^14^ It should be noted that this may not extrapolate to esophageal/gastroesophageal junction cancer radiotherapy, where the LCx dose is typically (and consistently) much higher than the heterogeneous exposure in lung cancer radiotherapy, depending on primary tumor and/or nodal station involvement (see subsection 2.3.4 below).

In a 2024 study of arrhythmia subtypes associated with distinct cardiac substructure dose exposure, Atkins et al. reported a 1% incidence of ventricular tachycardia/asystole at 2 years with a median time to onset of 20 months, which was associated with LMCA V50 Gy ≥0.5 cc.^16^ The authors observed an apparent clustering of cardiac event types, with nearly one-third of those who developed arrhythmia (especially ventricular tachycardia/asystole and bradyarrhythmia) also experiencing MACE, consistent with a possible mechanism of coronary injury resulting in myocardial infarction and the development of left ventricular scar-based re-entry loops or conduction blocks.

##### Coronary artery dosimetry in esophageal cancer

Building off the observations in lung cancer, studies in esophageal cancer reinforced the significance of coronary artery dose exposure and the risk of cardiac events. In a 2022 study of 355 patients treated with chemoradiotherapy between 2005 and 2015, Wang et al. reported a 4% rate of major coronary events at a median of 16 months, and this was correlated with LAD V30 Gy ≥ 10%, and LMCA mean ≥ 20 Gy was associated with mortality, both outperforming MHD or whole-heart V30 Gy.^11^ In a large 2023 study of 716 patients treated with definitive radiotherapy between 2010 and 2016, 16% developed grade ≥3 cardiac events at a median onset of 14 months.^12^ The investigators observed that LAD V30 Gy and LCx V45 Gy outperformed whole heart variables for the ischemic endpoint of acute coronary syndrome and/or congestive HF but performed similar to MHD for the composite endpoint of “any” grade ≥3 cardiac event. Cut-point analysis to identify optimal dose thresholds was not performed.

#### Cardiac dosimetry: ventricles and myocardium

##### Ventricle and myocardial dosimetry in breast cancer

Early cardiac substructure dosimetric data typically included the heart chambers only (ie, no coronary dosimetry), and LV outcomes may potentially be a surrogate for left coronaries when both are not studied. Studies by van den Bogaard et al. of 910 patients with breast cancer, with a median MHD of 2.4 Gy, observed a 3% occurrence of acute coronary events that better correlated with LV V5 Gy than MHD (no cut-point analysis was performed),^36^ but did not observe a correlation between LV or coronary dose predictors and LVEF decline.^44^ However, a subsequent study observed that LV V5 Gy was specifically associated with acute coronary events in patients with LAD atherosclerotic plaques (no cut-point analysis was performed).^41^

In 2019, the DEGRO expert panel recommended LV constraints of mean <3 Gy, V5 Gy <17%, and V23 Gy <5%,^46^ based on limited existing data. Specifically, Erven et al. evaluated 30 patients with breast cancer and observed differences in regional strain by echocardiography in LV segments receiving >3 Gy (median segmental dose).^57^ While LV V5 Gy <17% was not explicitly tested as a cut-point, this was the reported mean LV V5 dose among patients who did not experience an acute coronary event in the 2017 study by van den Bogaard et al.^36^ Lastly, ≥5% of the LV extending into the radiation fields (defined as >23-25 Gy exposure) was associated with perfusion defects on cardiac Single Photon Emission Computed Tomography (SPECT) imaging in a 2005 study of 114 patients by Marks et al.^58^ In echocardiography-based studies evaluating dose to the LV, subclinical LV dysfunction (including GLS deterioration) has been observed post radiotherapy, persisting at 6 and 12 months and found to be variably relating to LV (or LV segmental) dose.^49^^,^^59^

##### Ventricle and myocardial dosimetry in Hodgkin lymphoma and pediatric cancer

In the cardiac dosimetric analysis in pediatric cancer survivors, Bates et al. reported the 35-year cumulative incidences of CAD, HF, valvular disease, and arrhythmia were 4%, 4%, 1%, and 1%, respectively.^35^ There was no correlation with mean whole heart dose with cardiac diseases, though the mean of 5.0 to 9.9 Gy to the LV was associated with CAD, and that to the RV was associated with valvular disease.

##### Ventricle and myocardial dosimetry in lung and esophageal cancer

In a 2020 study by Jang et al., 258 patients with NSCLC treated with definitive chemoradiotherapy between 2008 and 2018 were analyzed for the relationship between heart chamber dosimetry, baseline ASCVD risk, and post-radiotherapy cardiac events.^60^ Overall, 11% of patients experienced cardiovascular events, and LV V60 Gy >0% correlated with acute coronary syndrome (4 events), though notably this relationship was only observed among patients with an ASCVD score >7.5%. These findings were consistent with Atkins et al.,^9^ which demonstrated that optimal cardiac dose constraints may differ by baseline CHD status. Specifically, while LAD V15 Gy ≥10% (or 1 cc^14^) was an independent predictor of MACE, particularly among those without CHD, in individuals with pre-existing CHD, LV V15 Gy ≥1% (vs <1%) was associated with double the risk of MACE (8% vs 4%, respectively). Together, these studies are suggestive that in patients with elevated baseline cardiovascular risk, there may be a greater susceptibility to myocardial injury or fibrosis.

Zhang et al. further parsed these dose relationships, evaluating the differential impact of LV myocardium vs LAD dose on incident HFrEF, HFpEF, and acute coronary events.^14^ The authors observed a 5% 2-year cumulative incidence of any grade ≥3 HF at a median onset of 17 months, including 4% and 2% 2-year rates of HFrEF and HFpEF, respectively, among patients without pre-existing HF. The 2-year cumulative incidence of any acute coronary event was 3%, with a median onset of 24 months, and was more common among those with pre-existing CHD at 12% compared to patients without CHD at 4%. On dosimetric analysis, while LAD V15 Gy ≥1 cc was specifically associated with acute coronary events (and not HFrEF or HFpEF), LV myocardium V20 Gy ≥11 cc was distinctly associated with HFrEF, but not HFpEF or acute coronary events. Notably, while no cardiac substructure dose predictors were significantly correlated with HFpEF, LAVI and the degree of lung resection were, suggesting that pre-treatment cardiovascular risk and postoperative pulmonary hemodynamics may be significant contributing factors.

A 2024 prospective study by Sławiński et al. in 43 patients with lung cancer observed a reduction in RV function (by free-wall strain) in 40% of patients within 3 months post radiotherapy and a correlation between mean RV dose and RVEF assessed immediately after radiotherapy.^61^ A similar prospective study by Umezawa et al. in 2023 evaluated 23 patients with esophageal cancer treated with chemoradiotherapy and identified LV 45 Gy > 2% as correlated with both myocardial fibrosis by cardiac magnetic resonance and grade ≥3 cardiac events.^62^

#### Cardiac dosimetry: atria, PVs, conduction system

##### Arrhythmias after thoracic radiotherapy

The data linking cardiac substructures and arrhythmia endpoints are largely derived from lung cancer studies. The incidence of arrhythmia events after radiotherapy ranges from 1% to 3% for pediatric cancer, Hodgkin lymphoma, and breast cancer,^13^^,^^17^^,^^35^^,^^63^ but more commonly occurs following lung and esophageal cancer radiotherapy, with rates of 5% to 20%.^7^^,^^8^^,^^15^^,^^17^^,^^64^, ^65^, ^66^ Most arrhythmias are supraventricular in etiology, with atrial fibrillation (Afib) occurring most commonly (5% to 11% in lung cancer,^15^, ^16^, ^17^^,^^65^ 8% to 20% in esophageal cancer^17^^,^^66^) and being associated with pre-existing cardiovascular risk.^8^^,^^16^^,^^17^ Atkins et al. additionally reported 2-year cumulative incidences of grade ≥3 non-Afib arrhythmias after radiotherapy, including atrial flutter (3%), other supraventricular tachycardia (2%), bradyarrhythmia (1%), and ventricular tachyarrhythmia/asystole (1%).^16^ Atrial flutter and other supraventricular tachycardias were not associated with baseline cardiovascular risk, while the remaining were.^16^

##### Cardiac dosimetry predicting atrial fibrillation

In a 2022 study of 560 patients with lung cancer, Kim et al.^65^ demonstrated that the sinoatrial (SA) node max dose (alongside RA and LA dose) was correlated with Afib and mortality. Notably, however, <15% of Afib ectopic foci originate near the SA node (the physiologic pacemaker)^67^ in the RA. Rather, most Afib foci (>80 to 90%) originate within the myocardial sleeves of the proximal PVs.^67^^,^^68^ These observations by Kim et al.^65^ (as PVs were not specifically delineated) may be explained by the surrogacy of the SA node (and atria) to the PVs given the anatomic proximity, rather than direct SA node injury. This is consistent with three subsequent studies demonstrating that PV dose variables outperform atria and SA node in predicting Afib.^15^, ^16^, ^17^ In a 2024 study of 420 patients treated with NSCLC treated between 2015 and 2020, Walls et al. demonstrated that left PV V55 Gy ≥2% and right PV V10 Gy ≥54% (but not SA node or LA dose) were correlated with the risk of grade ≥3 Afib.^15^ In the 2024 study of 701 patients with NSCLC by Atkins et al., PV V5 Gy ≥ 15 cc was associated with the risk of grade ≥3 Afib.^16^ Subsequently, Butler et al. analyzed a cohort of 539 patients with mixed thoracic cancers and identified PV max dose >40 Gy to be associated with the risk of grade ≥3 Afib.^17^

##### Cardiac dosimetry predicting non-Afib arrhythmias

In Atkins et al^16^ high-dose atrial exposure (LA/RA; top predictor, PV V55 Gy ≥31%) was linked to the risk of (non-Afib) supraventricular tachycardia. Moderate to high LCx dose was associated with atrial flutter (top predictor, LCx V35 Gy ≥1.8 cc), which may relate to its anatomic proximity to atypical atrial flutter pathways along the LA. Low to intermediate dose to the RCA, atrioventricular (AV) node, and RV were linked with bradyarrhythmia (top predictor, V25 Gy ≥1.9 cc), which may relate to direct AV node injury or indirect via the AV nodal branch of the SA nodal artery (>90% originate from RCA). Together, these studies emphasize the importance of understanding cardiac anatomy and substructure radiation exposure to identify plausible mechanisms of injury.

#### Cardiac dosimetry: heart base, great vessels

##### Heart base dosimetry

The heart base has been identified as a dose-sensitive cardiac region associated with mortality^69^^,^^70^ and composite cardiac events (Afib, HD, acute coronary syndrome)^71^ in lung cancer radiotherapy. Early work mapped the critical heart base area for survival around the RA (including the SA node and proximal coronaries),^69^ with subsequent studies describing this region centered around the LA,^70^ as well as on the origin of the left coronary artery and AV node.^72^ Notably, the voxel-wise image-based mining methodology in the above studies to identify dose-sensitive regions has been performed with survival as the endpoint (not cardiac events). In the study by Walls et al. linking heart base max dose to cardiac events, the structure was defined as a composite including the RA (including the SA node), superior vena cava, aortic root, LMCA, proximal LAD, and proximal RCA.^71^ The nature of this region including several substructures linked to a multitude of cardiac event types provides insight into its predictive power for composite cardiac events (ie, in Walls et al., Afib comprised 37% and HF 44% of the events), mortality, and as a potential surrogate for generalized significant cardiopulmonary dose exposure.

##### Pulmonary artery dosimetry

Pulmonary fibrosis after thoracic radiotherapy has been hypothesized to increase the risk of pulmonary hypertension (PH), although dosimetric data for this have been scarce. In a multi-institutional retrospective cohort of 848 patients with NSCLC, Atkins et al. reported (pre-print^73^) a 2-year cumulative incidence of any grade PH of 12%, which was associated with pulmonary artery V10 Gy (but not distal pulmonary vasculature, lung, or heart doses). Importantly, patients with PH after radiotherapy were more likely to develop RV systolic dysfunction and tricuspid disease.

#### Cardiac dosimetry: valves and other structures

##### Cardiac valve dosimetry

Among pediatric cancer survivors, the 35-year incidence of valvular heart disease (VHD) is 1% and associated with tricuspid valve and RV mean doses of 5.0 to 9.9 Gy.^35^ In a study of 56 Hodgkin lymphoma survivors, nearly one-third (32%) developed VHD, and VHD was associated with an LA V25 Gy >63% and LV V30 Gy >35% (mitral and aortic VHD), while RV V30 Gy >65% was associated with tricuspid VHD.^74^ In a 2015 case-control study of 89 patients with VHD (and 200 control patients), the risk of VHD increased significantly at valve doses >30 Gy, with a 30-year cumulative VHD risk of 6% to 12% for valvular doses between 30 to 40 Gy, and noting that with modern lower prescription doses, the rate may be closer to ∼1%.^75^ In lung and esophageal cancer, VHD dosimetry data are limited but suggest a 2% rate of grade ≥3 VHD after lung cancer radiotherapy.^8^

##### Pericardium dosimetry

The rate of grade ≥3 pericardial events is 3% to 7%^8^^,^^76^^,^^77^ in lung and esophageal cancer. Dosimetric data in esophageal cancer report a median onset of 7 months and associated with V30 Gy >66%, V40 Gy >55%, and V60 Gy >25%.^77^

##### Carotid artery dosimetry

While not specifically a cardiac outcome, ischemic stroke related to carotid artery disease following thoracic and head/neck cancer radiotherapy is well described.^1^ A retrospective study of 224 patients observed that baseline Framingham risk and mean radiotherapy dose to the carotid bulb (plus 2 cm) was associated with an increased risk of carotid stenosis.^78^ A follow-up study of 628 patients without known pre-existing carotid artery disease observed a 10-year actuarial rate of asymptomatic carotid artery stenosis of 30% and the rate of stroke or transient ischemic attack of 10%, respectively, after RT.^79^ On multivariable analysis, absolute carotid artery V10 Gy was significantly associated with asymptomatic carotid artery stenosis (>50% diameter).^79^

##### Other cardiovascular dosimetry

Cardiovascular autonomic dysfunction after thoracic radiotherapy has been described previously, although cardiac dosimetric studies are limited.^80^ In a retrospective cohort of 165 patients who underwent post-radiotherapy exercise stress testing (abstract^81^), a mean aortic arch dose and receipt of immunotherapy were associated with the risk of autonomic dysfunction, although MHD and sympathetic chain dose were not.

#### Ultra-hypofractionation

Biological radiation dose escalation with stereotactic body radiation therapy (SBRT) and hypofractionation regimens represents an increasing proportion of radiotherapy treatments, for both early lung cancers and pulmonary oligo-metastases, particularly with growing indications for metastasis-directed therapy. SBRT has historically been reserved for patients in whom the target is distant from central structures, including the heart; hence, the cardiac effects of SBRT regimens remain poorly characterized. Of the few published studies, most have not identified an association between heart/substructure dose and cardiotoxicity, although small sample sizes, missing baseline cardiovascular factors, restricted cardiac follow-up, or limited ranges of cardiac exposures were common study limitations.^82^, ^83^, ^84^ Furthermore, the basic radiobiology of the heart remains poorly understood, perhaps best illustrated by the emerging use of single-fraction ultra-hypofractionated radiation as a treatment for cardiac disease.^85^ Until higher-quality data are available, cardiac dose constraints from clinical trials should continue to be strictly applied.

#### Limitations

The aforementioned supportive evidence is largely retrospective and vulnerable to selection bias, as well as the risk of under- or over-capturing of cardiac event rates. Furthermore, there is incomplete adjustment for pre-existing cardiovascular risk, endpoint definitions are heterogeneous, systemic therapy exposures are inconsistent and not routinely accounted for, the degree of cardiac substructure inclusion varies widely, and cardiac substructure contouring may be sub-optimal or even inaccurate, all of which may impact observed dose relationships (see Supplemental Table for study-specific details). Finally, with the growing indications for metastasis-directed therapy in oligometastatic disease and the use of hypofractionated and SBRT regimens, a better understanding of the cardiovascular risks related to cardiac substructure exposure in this setting is warranted.

## Pillar 3

### Actionable strategies for clinical risk reduction

Highlights:

• Auto-segmentation tools reduce time and training barriers to facilitate routine incorporation of cardiac substructure dose constraints.
• Prioritizing a limited set of high-value substructures (eg, left coronaries and LV) with at least one optimization constraint improves feasibility and accelerates clinical translation.
• Use cardiac substructure dose as a risk signal, not a strict limit: Exceeding cardiac substructure dose constraints should be interpreted as a flag for cardiovascular risk (not a planning hard limit), prompting clinical risk communication. The necessary dose to the tumor should be prioritized/maintained, and recommended cardiac substructure dose thresholds should facilitate planning optimization with the lowest achievable exposure.
• Radiation oncologists should standardly report cardiac dose exposure to cardiologists, enabling effective dose-informed cardio-oncology surveillance.
• In the absence of substructure specific guidance in most societal statements, pragmatic, evidence-based dose constraints can be readily applied to inform cardiovascular risk in multi-disciplinary follow-up.

#### Cardiac substructure dosimetry: the evidence-to-practice gap

Implementation of cardiac substructure dosimetry remains challenging due to a combination of technical and practical barriers, including limited formal training (of radiation oncologists) in detailed cardiac anatomy, the time- and resource-intensive nature of manual substructure delineation (especially on non-contrast, often 4-dimensional, CT scans), and the absence of consensus high-value dose constraints or integration into national guidelines and clinical trials. This lack of practical cardiac dose-outcomes consensus has, in turn, hindered the development of actionable, dose-informed recommendations for cardiovascular risk surveillance within contemporary cardio-oncology guidance. Pillar 3 therefore focuses on translating the mechanistic insights from Pillar 2 into pragmatic, actionable approaches across the radiotherapy care continuum, from treatment planning to post-treatment cardiovascular risk modification and multi-disciplinary follow-up.

##### Radiotherapy-related cardiovascular risk in society statements and guidelines

Across contemporary cardio-oncology society guidelines and statements, radiotherapy-related cardiovascular risk is typically described in broad, qualitative terms that offer limited guidance for action (Table 1). Only the 2022 European Society of Cardiology cardio-oncology guideline proposes explicit dose strata, and these are restricted to the whole-heart mean dose.^86^ Other documents rely on phrases such as “high-dose chest radiotherapy,”^87^ “mediastinal radiotherapy,”^88^ or “significant thoracic radiotherapy,”^89^ without defining dose ranges or temporal factors. Similarly, National Comprehensive Cancer Network disease-site guidelines provide limited cardiac substructure risk guidance and primarily whole-heart dose metrics (with Hodgkin lymphoma as a notable exception with inclusion of comprehensive substructure dose limits) (Table 2). As a result, and due to a persistent lack of cardiac radiation dose data in the medical record, clinical recommendations frequently default to subjective clinical judgment rather than explicit dose-based triggers for cardiovascular intervention, leaving a substantial evidence-to-practice gap.

**Table 1:** Radiotherapy-Related Cardiovascular Risk Guidance Across Major Cardio-Oncology Society Documents

Author & Year	Society/Organization	Scope of RT-Related CV Guidance	RT-Related CV Screening Recommendations	Comments
Bloom 2025^103^	Heart Failure Society of America (HFSA)	Lists “prior chest RT” as one of several HF risk factors in cancer survivors	- Baseline CVRF assessment (all); TTE when indicated. - Closer monitoring with chest RT + anthracyclines or other cardiotoxic agents. - After chest RT, long-term follow-up for VHD, CAD, cardiomyopathy, and pericardial disease.	- No RT dose, era, or exposure specificity; no RT-based decision thresholds - Surveillance is based on qualitative risk
Blaes 2025^104^	American College of Cardiology (ACC)	RT described as cardiotoxic exposure	- Annual CVRF screening - After RT, TTE at 1-5 y; ongoing periodic imaging, especially if >30 Gy or + anthracycline. - Noninvasive CAD screening at 5 y post-RT and every 5 y for “higher-risk” RT exposures.	- Survivorship Expert Panel - No additional dose specifics (refers to ESC cardio-oncology risk models)
Leong 2024^89^	American College of Cardiology (ACC)	Refers to ESC cardio-oncology risk models	- No specific RT-linked surveillance	- No new dose thresholds or substructure level detail; “significant thoracic RT” undefined; referral guidance framed as multi-disciplinary discussion
Addison 2023^105^	American Heart Association (AHA)	RT (left-sided breast) only mentioned as a risk factor for CVD	- No specific RT-linked surveillance - Highlights that high-risk patients often have reduced access to preventive CV assessment. It does not specify imaging frequency, biomarker monitoring, or RT-linked surveillance.	- No RT details - Statement focuses on equity and disparities framework across cardio-oncology
Lyon 2022^86^	European Society of Cardiology (ESC)	- Defines MHD-based risk categories (<5, 5-15, 15-25, ≥25 Gy); if MHD unavailable, utilize prescription dose - Recommends minimize cardiac RT dose and optimize technique	- Assess baseline CV risk before RT - Lifetime surveillance after chest RT - Imaging (echo, stress testing, CTCA) based on symptoms and risk factors - No RT dose tied to screening	- Only statement/guideline with explicit (whole heart; MHD) dose thresholds - Cardiology referral language subjective
Herrmann 2022^106^	International Cardio-Oncology Society (IC-OS)	RT mentioned only as a risk factor for myocarditis and vascular injury	- Not a screening guideline - No RT-specific screening recommendations, dose guidance, or surveillance intervals	- No RT details - Defines CV toxicities of cancer therapies; modality-agnostic
Beavers 2022^107^	American Heart Association (AHA)	Focus on cardio-oncology drug-drug interactions	- No specific imaging intervals or RT-specific surveillance protocols are defined.	- No RT details - RT (to the chest wall) only mentioned as cardiomyopathy risk factor
Bose 2022^108^	American College of Cardiology (ACC)	- No explicit RT dose thresholds adopted by consensus. - Panelists disagreed on RT dose requiring screening: 56% after any chest RT, 33% if MHD >15 Gy, 10% if MHD >30 Gy.	- Consensus for lifelong TTE, cardiology referral for EF ≤50% or diastolic dysfunction - Disagreement on advanced imaging and biomarkers - Radiation influences screening frequency but not through defined dose bands	- Consensus on screening and managing cardiomyopathy in CCS - Provides no numeric RT dose thresholds - RT risk language remains qualitative
Mitchell 2021^19^	International Cardio-Oncology Society (IC-OS)	Reviews dose-response for CAD, VHD, pericardial and myocardial disease; emphasizes the importance of coronary and other cardiac substructures	- Most detailed RT-specific screening algorithm (Figure 1, pg 4). - Baseline CVRF assessment: H&P, ECG, TTE when indicated; review CT for coronary/aortic calcification; optimize CVRF. - After thoracic RT: yearly CV exam; consider ischemic testing; TTE at 6-12 months for high-risk patients; ongoing TTE surveillance. - After H&N RT: carotid ultrasound every 5y (earlier in high-risk). - After abd/pelvic RT: clinical exam for PAD, renal function; imaging when indicated.	- Excellent conceptual RT-related CV risk detail - No dose-related risk groups or referral triggers - Emphasizes early detection of atherosclerosis (CAC, CTCA) and personalized surveillance.
Curigliano 2020^88^	European Society for Medical Oncology (ESMO)	Identifies “high-dose mediastinal RT” and combined anthracycline + RT as major risk factors	- Baseline CVRF assessment - Screening for pts receiving L-sided or mediastinal RT (particularly with cardiotoxic systemic agents) based on risk factors and symptoms	- “High-dose” and “mediastinal” not defined - No specific dose ranges or cardiac exposure criteria
Armenian 2017^87^	American Society of Clinical Oncology (ASCO)	- Defines high risk as 1) high-dose chest RT ≥ 30 Gy with the heart in field, or 2) <30 Gy when combined with anthracycline - Minimize mediastinal exposure	- Baseline H&P, CVRF assessment, TTE - During therapy: H&P, TTE if symptoms - After therapy: no specific schedule; risk-based	- Binary prescribed (not cardiac) dose threshold - “Heart in field” is subjective
Armenian 2015^109^	Children's Oncology Group (COG)	- Historical COG dose categories: 1) higher risk with ≥35 Gy mediastinal/chest RT, 2) moderately increased risk with 15-35 Gy, 3) lower but non-zero risk with <15 Gy	- Periodic TTE based on RT risk tired and anthracycline exposure - Earlier/more frequent screening with RT + anthracycline	- No MHD-based dose thresholds - Uncertainty in minimum RT dose meriting surveillance
Lancellotti 2013^110^	European Association of Cardiovascular Imaging (EACVI) and the American Society of Echocardiography (ASE)	- Describes RT dose-dependent risk (but no dose thresholds) - Identifies >30 Gy anterior/left chest RT as historically associated with increased CV risk	- Baseline and periodic imaging (primarily TTE) with modality tailored to suspected pathology (e.g., CT for coronaries, MRI for fibrosis, TTE for valves, etc.)	- No RT dose thresholds

ACC = American College of Cardiology; AHA = American Heart Association; ASCO = American Society of Clinical Oncology; ASE = American Society of Echocardiography; CAD = coronary artery disease; CCS = childhood cancer survivors; CV = cardiovascular; CVD = cardiovascular disease; CVRF = cardiovascular risk factor; EACVI = European Association of Cardiovascular Imaging; ESMO = European Society for Medical Oncology; ESC = European Society of Cardiology; HF = heart failure; HFSA = Heart Failure Society of America; IC-OS = International Cardio-Oncology Society; IMRT = intensity-modulated radiotherapy; MHD = mean heart dose; N/A = not applicable; RT = radiotherapy; TTE = transthoracic echocardiography.

**Table 2:** Cardiovascular Guidance and Cardiac Substructure Dose Considerations Across NCCN Thoracic and Mediastinal Disease-Site Guidelines

Disease site	NCCN version	Scope of Cardiovascular Guidance	Cardiac Substructure Constraints (Yes/No)	Comments
Breast	v5.2025	Limited; CV risk is *acknowledged* (esp. left-sided RT), use of heart-sparing techniques emphasized	No	No numeric heart or substructure thresholds; no CV surveillance guidance.
Hodgkin lymphoma	v1.2026	Moderate (most detailed of the sites); explicit attention to late CV risk from mediastinal RT (esp. in young patients and long-term survivors)	Yes; dose limits provided for^a^^b^: total coronaries, LAD, LCx, RCA, all valves, LV, RV	Only NCCN site with explicit cardiac substructure constraints. Generalized post-treatment echo considerations.
NSCLC	v1.2026	Limited	No	Whole-heart constraints only; no CV surveillance guidance.
SCLC	v2.2026	Limited	No	No whole heart constraints or CV guidance.
Esophageal/GEJ	v8.2025	Limited; encourages conformal/advanced techniques (IMRT, proton) to reduce cardiopulmonary dose.	No	Whole-heart constraints only; notation that heart dose should be minimized (i.e., via IMRT/protons); no CV surveillance guidance.
Thymic tumors	v1.2026	Limited; note the importance of minimizing cardiac dose given younger age and long survivorship.	No	Reference is made to NSCLC normal tissue constraints; conformal or proton techniques should be considered.

CV = cardiovascular; esp. = especially; GEJ = gastroesophageal junction; IMRT = intensity-modulated radiotherapy; LAD = left anterior descending coronary artery; LCx = left circumflex coronary artery; LV = left ventricle; NSCLC = non–small cell lung cancer; RCA = right coronary artery; RT = radiotherapy; RV = right ventricle; SCLC = small cell lung cancer.

a LAD and LV (and mean heart) dose limits noted to be most important constraints.

b Coronary dose constraints extrapolated from lung cancer studies (Atkins et al. 2025 *JAMA Oncol*).

#### Bridging the cardiac dosimetry evidence-to-practice gap

##### Cardiac substructure contouring

Although several contouring guidelines exist for cardiac substructures, including coronary arteries,^90^^,^^91^ conduction system,^92^ and PVs,^93^ manual segmentation can be laborious. A growing number of cardiac auto-segmentation algorithms have been reported,^94^, ^95^, ^96^ and commercially available auto-segmentation tools increasingly include cardiac substructures (e.g., TheraPanacea, Paris, France; Limbus AI, RAD formation; MIM Software). These automated segmentation tools can reduce the implementation barrier to cardiac-sparing radiotherapy techniques.

##### Practical implementation of high-value cardiac substructure dose constraints

**Figure 3:**
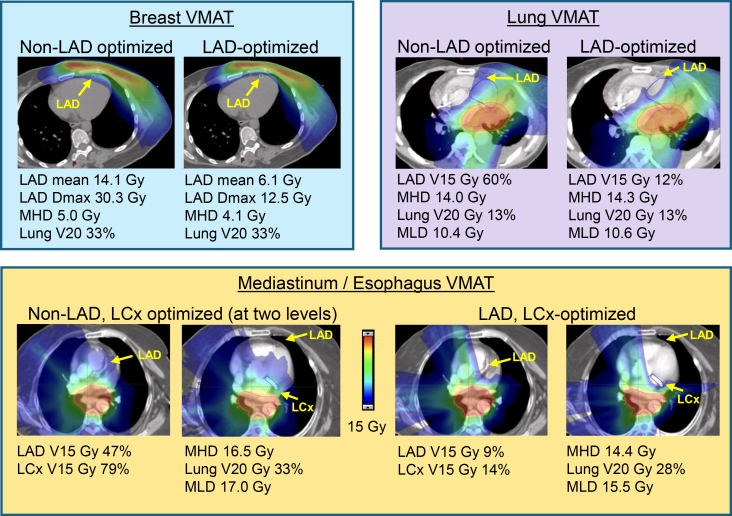
Radiotherapy Planning with Active Coronary Dose Sparing In these plans, left coronary dose was actively constrained (breast plan: goal left anterior descending [LAD] mean <10 Gy, maximum <20 Gy; other plans: goal LAD V15 Gy <10%, left circumflex (LCx) V15 Gy <14%) with at least moderate priority in the optimizer and tumor coverage/dose was maintained at equivalent levels. Dose color wash lower level set at 15 Gy. All coronary-optimized plans preserved/maintained prescribed dose to tumor. MHD = mean heart dose; MLD = mean lung dose; VMAT = volumetric modulated arc therapy.

Even with manual contouring, however, radiation oncologists should be empowered to prioritize a limited set of high-value cardiac substructures and constraints. Based on the totality of cardiac outcome-based studies (Figure 1, Supplemental Table) and the potential morbidity of ischemic and HF outcomes, contouring the left coronary arteries and the LV (or LV myocardium) with at least one dosimetric optimization goal (or dose constraint) is practical and feasible. In Table 3, we describe a practical approach to implementing cardiac substructure dose constraints and to radiotherapy planning (see Supplemental Figure for example high-value contours). In Figure 3, we illustrate practical examples of planning optimization including the coronary arteries while maintaining effective tumor dosing. These recommendations are consistent with contemporary radiotherapy planning studies demonstrating the feasibility of cardiac substructure dose optimization.^97^, ^98^, ^99^

**Table 3:** High-Value Cardiac Substructure Dose Constraints and Practical Radiotherapy Planning Considerations

Cardiac substructure	Associated cardiac outcome	Practical contouring tips	Practical dose constraints for consideration^c^	Planning considerations
LMCA^a^/LAD^a^^b^	Acute coronary events, MACE	- LMCA is typically only on 1-2 slices, combine with LAD structure for simplicity	- V15 Gy <10%/1 cc - Mean <5-10 Gy (breast)	- In curative intent treatment, tumor/PTV coverage is typically *priority* over cardiac substructures - Consider lung and heart (substructures) at similar initial priority level during optimization; if lung is exceeding tolerance, optimize with lung >heart - When tumors are anatomically close to a CA (e.g., LCx for esophageal cancer), moderate isodose (i.e., V15 Gy) sparing is not typically feasible and ALARA is recommended (e.g., Dmax <105% Rx) - For right-sided lung cancer or right-breast with RNI treatment, low-to-moderate dose (10-15 Gy) can often be carved off CAs with little to no cost/tradeoff
LCx^a^^b^	Acute coronary events, MACE	- Can be challenging to identify, contour within the atrioventricular groove to as a practical estimate	- V15 Gy <14%/1 cc	
LV, LV myocardium	Acute coronary events, MACE, heart failure, subclinical cardiac dysfunction	- Include the interventricular septum in LV contour - LV myocardium contour excludes blood pool (to estimate on non-contrast CT, use 1 cm contracted margin)	- LV V15 Gy <1% - LV Mean <3 Gy (breast) - LV myo V10 Gy <11 cc	

ALARA = as low as reasonable achievable; CA = coronary artery; Dmax = maximum dose; LA = left atrium; LAD = left anterior descending coronary artery; LCx = left circumflex coronary artery; LMCA = left main coronary artery; LV = left ventricle; MACE = major adverse cardiac event; myo = myocardium; RNI = regional node irrigation.

a Use 7 to 8 mm brush to account for cardiac and uncertainty related to respiratory motion and/or degree of visualization; 4 to 5 mm is reasonable in the setting of breath-hold ± intravenous contrast depending on degree of visualization.

b If combining LMCA + LAD + LCx (i.e., TotalLeft CA), use V15 Gy <2.5 cc.

c For non-breast sites, these constraints are based on conventional fractionation (eg, 1.8-2.0 Gy per day) and not intended for hypofractionated regimens, including stereotactic ablative radiotherapy.

While the preferred planning approach for optimal cardiac-sparing is not precisely known, in left-sided breast radiotherapy with regional nodal irradiation, there are often differences in whole heart radiotherapy dose distributions between intensity-modulated radiotherapy (IMRT) and 3-dimensional conformal radiotherapy (3D-CRT), even with similar MHDs, with increased volumes of lower heart doses with IMRT vs increased volumes of higher heart doses with 3D-CRT. Thus, the MHD-associated linear risk with ischemic heart disease^5^^,^^8^^,^^38^ may not be the same with breast IMRT compared to tangential 3D-CRT.^100^ Using IMRT in these patients, if LV and LAD constraints are used during planning optimization, high-dose regions in these substructures are often lower than those of 3D-CRT plans.^101^ In lung cancer, IMRT has been associated with reduced risk of MACE compared to 3D-CRT.^8^^,^^9^ In cases where planning techniques may yield discordance between the whole heart and coronary dose, the risk of MACE has been observed to be lower among those with isolated low LAD dose (independent of MHD).^34^ Furthermore, while modern data including the use of proton radiotherapy has shown significantly lower MHD and LAD dose exposures,^55^ the radiobiologic implications of proton range uncertainty at the heart surface, including at the LAD, are not yet known, but typical planning approaches prioritize reduction in MHD.

##### Considering cardiac dose-constraints as risk thresholds

It is acceptable to exceed the suggested cardiac substructure dose constraints if necessary to achieve adequate tumor radiotherapy dose coverage. However, the goal should remain to *actively* minimize cardiac substructure (i.e. LAD, LV) dose exposure through intentional optimization parameters and not simply to rely on passive avoidance. When these dose thresholds are exceeded, this should alert the radiation oncologist to an increased cardiovascular risk (ie, higher risk of acute coronary events with elevated left coronary dose or Afib with elevated PV dose). Importantly, given the opportunity for standard-of-care clinical cardiovascular risk factor mitigation approaches, it is essential to communicate the presence of any noted coronary artery calcifications and radiotherapy-related cardiovascular risk to the primary care and/or cardiology team.

##### Radiation oncology and cardiology/cardio-oncology crosstalk

**Figure 4:**
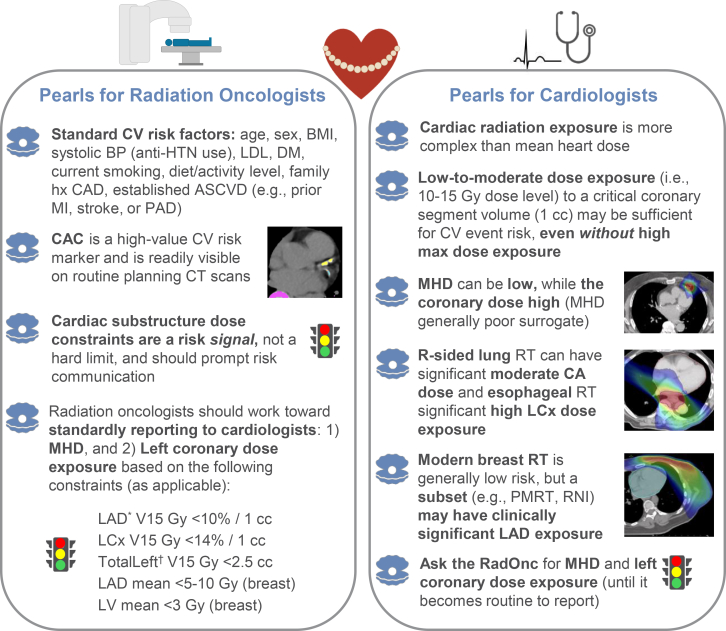
Practical Pearls for Radiation Oncology and Cardiology Crosstalk ∗Reasonable to include the left main coronary artery (LMCA) with the LAD contour as a single volume for simplicity, given the LMCA typically spans 1 to 2 slices. This avoids unaccounted-for or unconstrained dose to the LMCA if only the LAD is contoured/constrained. ^†^TotalLeft includes LMCA + LAD + LCx coronary arteries. ASCVD = atherosclerotic cardiovascular disease; BMI = body mass index; CA = coronary artery; CAC = coronary artery calcium; CAD = coronary artery disease; CV = cardiovascular; DM = diabetes mellitus; HTN = hypertension; hx. = history; LAD = left anterior descending coronary artery; LCx = left circumflex coronary artery; LDL = low-density lipoprotein; LV = left ventricle; MHD = mean heart dose; MI = myocardial infarction; PAD = peripheral artery disease; PMRT = post-mastectomy radiotherapy; R = right; RNI = regional nodal irradiation; RT = radiotherapy; SBP = systolic blood pressure.

The current cardio-oncology guideline landscape (Table 1) represents an important opportunity to move from qualitative descriptors of “significant” heart radiation exposure toward actionable, substructure-based criteria that better reflect contemporary planning techniques (ie, inverse-planning models that rely on specified, weighted optimization criteria) and the emerging dosimetric evidence base. Ultimately, cardiologists cannot optimally perform dose-informed cardiovascular risk surveillance until cardiac radiation dose reporting is standardized. To aid in crosstalk between radiation oncology and cardiology, radiation oncologists should report to cardiologists and/or primary care physicians, at minimum, MHD and left coronary artery dose exposure (Figure 4, Table 3). The reporting of coronary artery dose exposure to the clinical care team should include recommended constraints for reference (ie, mean or V15 Gy for left coronaries, LV) or even qualitative assessment of dose (eg, none/minimum vs moderate/high). If the recommended coronary/LV constraints (Figure 4, Table 3) are exceeded, contemporary evidence supports considering this dose exposure a clinically significant risk and then following radiotherapy-specific screening/surveillance strategies (Table 1), the most detailed of which are described by Mitchell et al. in the International Cardio-Oncology Society consensus guidelines.^19^

##### Future directions

While the above steps aid practical implementation based on the current best evidence, continued work is needed, particularly standardized dosimetry reporting and cardiovascular risk assessments in prospective datasets. Several important prospective studies remain ongoing, including those integrating cardiac imaging and biomarkers with cardiac substructure dosimetry, such as CLARITY (NCT04305613; accrual completed)^102^ and HEARTS (HEARTS Trial for Thoracic Cancers; NCT07132918), a phase II interventional trial of cardiac substructure-sparing magnetic resonance-guided adaptive radiotherapy. Additional prospective studies evaluating cardiac substructure dose outcomes include LOCATION MATTERS (Improving the Success Rate for Thoracic Radiotherapy Through Specific Cardiac Substructure Dosimetry; Locarion Matters; NCT06361784), SATIATION (Impact of Dose to Cardiac Substructures on Survival in Patients With ESophageal Cancer Treated With Radiotherapy of Chemoradiotherapy; NCT05996276), ACCOLADE (Avoiding Cardiac Toxicity in Lung Cancer Patients Treated With Curative-intent Radiotherapy; NCT03645317), and RACCOON (Radiotherapy for Thoracic and Breast Cancer and the Related Cardiotoxicity Following Treatment) NCT04674501), as well as large registry-based efforts such as the UK RAPID-RT study.

## Conclusions

Radiotherapy remains a cornerstone of oncology care. As advances in cancer therapy continue to improve survival, the cardiovascular impact of radiotherapy is of increasing concern. There is no established “safe” radiotherapy dose to the heart, and optimal cardiac substructure dose-sparing in the modern era must be intentional and evidence-informed. In this review, we outline the evidence base for associations between heart substructure radiotherapy dose and cardiac outcomes and offer pragmatic, actionable strategies to narrow the evidence-to-practice gap for dose constraints in modern radiotherapy. Efforts to optimize education for the cardio-oncology care team through decision-support tools, website- and application-based resources, and risk calculators are warranted. Further work is needed to incorporate cardiac substructure dose-exposure thresholds into actionable cardiovascular screening and surveillance strategies and to identify patients who may benefit from advanced radiotherapy technologies.

## Funding Support and Author Disclosures

Dr Atkins has received funding from the Garber Award for Cancer Research, the Developmental Award for Investigator Initiated Trials Using Advanced Imaging Methods. She has also received funding from Varian Medical Institutions (for her institution). Dr Bergom has received funding from NIH R01HL147884 and the Radiation Oncology Institute. Dr Walls holds a British Heart Foundation Project Grant for radiation cardioprotection research. Dr Karlstaedt Dr Atkins is supported by R01-HL-177461 and the Smidt Heart Institute. Dr Atkins reports honoraria from OncLive, Revolution Medicines speaker work for Ion Beam Applications; and; consulting for United Nations UNSCEAR CircuDis. Dr Walls reports speaker and consulting work for AstraZeneca. Dr Mitchell reports consulting for Alnylam, AstraZeneca, BridgeBio, and Pfizer. Dr Mak reports consulting with AstraZeneca, ViewRay, Novartis, Sio Capital Mgmt, Varian Medical Systems; advisory board with ViewRay, AstraZeneca; grant funding from AstraZeneca, ViewRay. Dr Bergom reports consulting for United Nations UNSCEAR CircuDis and the American College of Cardiology Foundation. All other authors have reported that they have no relationships relevant to the contents of this paper to disclose.
