# Supplementary material for: Heart Substructure Radiation Dose and Cardiac Outcomes: Contemporary Evidence and Actionable Opportunities: JACC CardioOncology State-of-the-Art Review
Source: JACC CardioOncol. 2026 Jun 16;8(3):211–30. doi: 10.1016/j.jaccao.2026.04.009 (PMC13282832; doi:10.1016/j.jaccao.2026.04.009)
Supplement: Supplemental Figure and supplemental Table [file mmc1.docx]

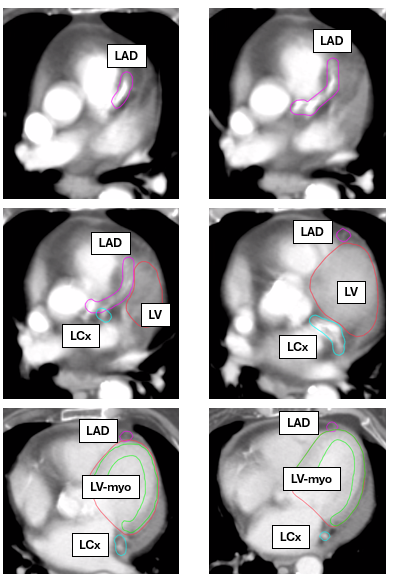
**Supplemental Figure. Example Contours of High-Value Cardiac Substructures.** Patient with locally advanced lung cancer with contrast-enhanced 4-dimensional CT simulation. Contours shown on average intensity projection. Left main coronary artery (LMCA) in combination with left anterior descending coronary artery (LAD; magenta) as single structure, left circumflex (LCx; cyan), left ventricle (LV; red), left ventricular myocardium (LV-myo; green).

**Supplemental Table. Cardiac Substructure Dosimetry Studies with Cardiovascular Event Outcomes.**

| Lead author / Year | Cancer Site | Study design; Sample Size (n) | Follow-up (median) | RT Years; RT Technique; Prescription | Baseline CV Risk (median age, reported CV risk factors) | Cardiac Outcome(s) | Cardiac Outcome Incidence/Rates | CS Analyzed; Dose Parameters Tested | Significant CS Predictors; Main Findings/Comments |
| --- | --- | --- | --- | --- | --- | --- | --- | --- | --- |
| Kang 2025^1^ | Breast | RCT; 693 | 6.5 y | 2015-2018; 50% 3D-CRT, 50% IMRT; 50.4 Gy/28 fx | 53y (med.), 7% DM, 21% HTN, 2% smoking, 4% famHx CVD | MCE (MI, UA, CA revasc., hospitalization due to cardiac arrhythmia) | 2% MCE | WH, LA, LV, total CA; Mean | None; ↑21% in MCE per Gy MHD; LV exhibited marginal significance in UVA analysis |
| Lamy 2025^2^ | Breast | Prospective; 138 | 2 y | 2017-2019; 36% 3D-CRT, 65% IMRT; 40-50.4 Gy/15-28 fx | 58y (med.), 21% BMI ≥30, 18% HTN, 17% current smoker, 6% DM, 15% statins | GLS (LV, RV, LA), LV EDVi, LV ESVi, mass, concentric remodeling, RVSVI, RVEF, RVCO | 3% ↓ mean LVEDVi and LVESVi; LVEF stable; 4% ↑ LV concentric remodeling; RVSWI, RVEF, RVCO ↓ | WH, LV, RV; Mean, max, V1 to V5 Gy | LV mean; 17% of pts w/ persistent ↓ in LV GLS, associated w/ higher mean LV doses |
| Nesseler 2025^3^ | Breast | Retrospective; 85 | 1.6 y | 2018-2023; 92% 3D-CRT, 8% IMRT; 42.6-50 Gy/16-25 fx | 50y (med.), 5% current tobacco, 19% HTN, 17% HLD, 18% CAC>0 | Any Gr≥2 CTCAEv5 (CV death, MI, PCI, UA, HF/CM, EF decline, VHD, dysrhythmias/conduction abnormalities, pericardial disease); ECG, TTE measurements | 11% Gr≥2 CE (med. 7.3mo; 1y 10%); ECG: ↑QTC prolongation ≥450 ms; ↑HR (resolved post-tx) | WH and LAD; Mean | LAD mean; LAD mean dose associated w/ ↑CE in TNBC w/ chemoIO |
| Guo 2025^4^ | Breast | Retrospective; 174 | 9.8 y | 2005-2014; 94% 3D-CRT, 3% IMRT; dose (NR) | 47y (med.), 100% F, 3% current smoking, 8% DM, 21% HTN, 8% prior Gr≥3 CE | Any Gr≥3 CTCAEv5, grouped: 1) ischemic events, 2) constrictive events, 3) valvular events, 4) conduction abnormalities | 8% CE | WH, LV, RA, LAD, LCx, LMCA, RCA; mean, max | None; No significant CS dose associations |
| Berlin 2025^5^ | Breast | Prospective; 303 | 5.1 y | 2010-2019; 95% photon, 5% PBT; med. 52.6 Gy/28 fx | 52y (med.), BMI 28 (med.), 36% any tobacco, 10% DM, 33% HTN, 22% HLD, 2% MI/CAD, 7% HF, 3% arrhythmia, 13% statins | TTE changes: LVEF, longitudinal strain, circumferential strain, E/e', ventricular-arterial coupling, RV fractional area change (as a measure of global RV systolic function) | At 5y, ↑ in LVEF (52% to 54%); ↓ circumferential strain (24% to 21%) | WH, LA, RA, LV, RV, LAD; Max, mean | LAD Dmax, LAD mean ≥10 Gy, LV mean ≥3 Gy; LAD max associated w/ modest ↓LVEF, longitudinal strain, circumferential strain, and E/e’; using DEGRO limits, LAD mean ≥10 Gy, mean LV ≥3 Gy (but not LV V5 Gy ≥17%) were associated w/ CV dysfunction |
| Spoor 2025^6^ | Breast | Retrospective; 5,808 (4,305 dev.; 1,503 ext.) | 6.2 y (dev.), 7.6 y (ext.) | 2005-2015; 3D-CRT or IMRT; dose (NR) | Dev. vs ext. cohort: 59 y vs 54y, former/current smoker 57% vs 20%, IHD 4% vs 1%, DM 10% vs 5%, HLD 12% vs 13%, HTN 30% vs 20% | ACE (MI, PCI, and IHD mortality) | Development v external: 3% v 1% ACE | WH, LA, RA, LV, RV, LAD; Mean, V1-10 Gy (1 Gy bins), V15, V20, V30 Gy; D1-5, D10, D50, D90, D95-D100 | LV-V5; MHD, LV-V5, + CAC associated w/ ACE; external validation failed (limited sample size/events). |
| Populaire 2025^7^ | Esoph. | Retrospective; 106 (77 dev.; 29 validation) | NR | 2015-2023; IMRT (45-50.4 Gy/25-28 fx) | 66y (med.), 82% M, 18% CCI-heart+ | Any Clavien-Dindo Gr≥2+ CE (cardiac arrest, MI, atrial or ventricular dysrhythmia, CHF, pericarditis); EF, LV GLS | 31% CE post-op (all atrial arrhythmias); 19% CE | LV; Mean, V5-40 Gy in 5Gy bins | LV V10 Gy; VBA-based identification of LV V10Gy as associated w/ CE |
| Lee 2025^8^ | Liver | Retrospective; 1,893 (1,473 dev.; 420 validation) | 2.5y | 2011-2022; SBRT; ≥30 Gy (≤5 fx) | Development: 64y (med.), 76% M, 29% current smoker, 35% HTN, 29% DM, 20% HLD, 3% CAD | Any Gr≥3 CTCAEv5 (incl. CAD, VHD, arrhythmia, pericarditis, HF) | Development: 6% MACE, 4% CAD (med. 23 mo.); Validation: 7% MACE, 4% CAD (med. 19 mo) | WH, chambers, ascending aorta, PA, IVC; Mean, max, V5-V45 in 5Gy bins (%) | WH V5; LA, LV RV, RA (mean) and LA, RA max; Models with WH V5 and chambers performed comparably (WH V5 selected for validation) |
| Zhang 2025^9^ | Lung | Retrospective; 748 | 5.5y | 2001-2014; 78% 3D-CRT, 22% IMRT; med. 64 Gy | 65y (med.), 51% M, 90% current tobacco 40%, 50% HTN, 48% HLD, 14% DM, 14% arrhythmia, CHD 36% | Gr≥3 CTCAEv4 HF/CM, HFrEF, HFpEF; ACE (UA, CA revasc., CV death) | 10% Gr≥3 HF, 2y 5%, med. 1.4y; 7% ACE; 2yr 3%, med. time 2y | LAD and TotalLeft (LMA, LAD, LCx), LV, LVmyo; V5, V10, V15Gy (cc) | LVmyo V10Gy ≥10.7 cc (HFrEF), LAD V15 Gy ≥1 cc, TotalLeft V15 Gy ≥2.5 cc (ACE); LVmyo V10 Gy associated w/ ↑HFrEF (but not ACE); LAD V15 Gy associated w/ ↑ACE (but not HFrEF); baseline LAVI and extent of lung resection associated w/ HFpEF. |
| Atkins 2025^†10^ | Lung | Retrospective; 850 | 5.5y (disc.), 3.9y (validation) | 2003-2014; 71% 3D-CRT, 29% IMRT; med. 61 Gy | 65y (med.), 50% M, 36% current tobacco, 52% HTN, 49% HLD, 16% DM, 35% CHD | Any grade PH, CTCAE Gr≥2 TV disease, RVSD, thrombotic event | 2y PH 12% at med. 1.7y; 6% Gr≥3 PE, 3% Gr≥2 TV disease, 11% Gr≥2 RSVD | WH, chambers, lungs, lung vessels, PA; mean, max, V5-60 Gy in 5Gy bins | PA V10Gy ≥55 cc; PA V10Gy associated w/ PH; among PH+ after RT, RVSD and tricuspid disease more common |
| Nesseler 2025^†11^ | Mixed | Retrospective; 165 | 2.3y | 2009-2024; NR | 68y (med.), 87% F, 49% HTN, 12% DM, 5% CHD, 15% statins | CV autonomic dysfunction | 2y (9%), 5y (18%) | WH, aortic arch, sympathetic trunk; mean, max, V5, 15, 30Gy (cc) | Mean aortic arch; Mean aortic arch dose associated with ↑autonomic dysfunction (MHD, sympathetic chain dose were not) |
| Qiao 2025^12^ | Mixed | Retrospective; 984 (701 disc. 701; 283 validation) | 5.2y | 2003-2021; Discovery (77% 3D-CRT, 23% IMRT; med. 60 Gy/30 fx) | Discovery: 65y (med.), 49% F, 92% any tobacco, 52% HTN, 49% HLD, 14% DM, 36% CHD | MACE (UA, HF, MI, CA revasc., CV death) | 10% MACE; med. time 1.6y | Chambers, CAs (LMCA, LAD, LCx, RCA, PDA); Mean, max, V5-60 Gy in 5Gy bins | LADV15 Gy, LCx V15 Gy; LAD V15 Gy, LCx V15 Gy, RAVI, LAVI associated w/ ↑MACE |
| Guthier 2025^†13^ | Mixed | Retrospective & Prospective; 560 (train.); 353 (valid.); 3399 (retro. longitudinal); 1,386 (prospective) | 5.5y (training) | 2003-2014 (training); Training (76% 3D-CRT, 24% IMRT; med. 66 Gy/33 fx) | Training: 65y (med.), 51% M, 92% any tobacco, 52% HTN, 15% DM, 29% CHD | MACE (UA, HF, MI, CA revasc., CV death) | 2y 5% (validation) | LAD, LCx, total CA; V15 Gy, mean | LAD V15 Gy ≥10%; Validated DL-system accurately segments cardiac CS, enables large-scale surveillance and POC alerts |
| Carpenter 2025^14^ | Head & Neck | Retrospective; 628 | 4.8y | 2000-2020; 91% IMRT; 75% conventionally fractionated | 61y (mean), 80% M, 56% smoking, 46% HTN, 34% HLD, 15% DM, 10% CAD | Asymptomatic CAS (≥50% reduced luminal diameter), symptomatic CAS (stroke or TIA) | 10-y: 30% asymptomatic, 10% symptomatic | Carotid artery; mean, min, max, V10-V70 (10 Gy bins) | Carotid V10 Gy; absolute V10 Gy (mL) was associated with asymptomatic CAS |
| Honaryar 2024^15^ | Breast | Prospective; 113 | 2y | 2015-2017; 3D-CRT; 50 Gy/25 fx | 58y (mean), 24% HTN, 5% DM, 49% smoking, 31% HLD | LVEF and/or GLS reduction | 44% cardiac dysfunction | Whole heart, LV, LAD, LCx; Mean, D2, V2 | LV V2 >36%, LCx mean >1.4 Gy; LAD and LCx dose were associated with asymptomatic cardiac dysfunction; subclinical endpoint |
| Quirk 2024^16^ | Breast | Retrospective; 3313 | 7y | 2012-2018; 3D-CRT; 81% 42.5Gy/16 fx | 60y (med.), HTN, HLD, DM, CKD, CHD (% NR) | MACE (MI, UA, HF, CA revasc.) | 4% 10-y (med. 7y) | WH, LAD; Mean, max | LAD V1Gy ≥9cc; modern cohort, LAD outperformed MHD |
| Perman 2024^17^ | Breast | Case-control; (109 cases, 59 controls) | >10y | 1958-1992; 72% orthovoltage, 24% megavoltage; dose (NR) | 62y (med.), 3% CHD, 9% smoking, 3% DM, 25% HTN | IHD | IHD | WH, RCA, LAD; Mean, max | RCA Dmax, LAD Dmax; Linear relationship between LAD and RCA Dmax w/ long-term fatal IHD; historic RT era |
| Jin 2024^18^ | Esoph. | Retrospective; 350 | 1.87y | 2013-2022; IMRT; 36-40 Gy/20 fx | 70y (med.), 70% M, 50% smoking, 33% EtOH, 25% HLD, 31% HTN, 7% DM, 15% CHD | Any Gr CE, Gr≥3 (CAD, HF, VHD, PCE, arrhythmia) | 57% any grade CE (3y 43%), 7% Gr≥3 | Pericardium, RA, SAN, LAD, RCA, LA, LV; Mean, max V5-60 Gy (%) | LAD V20 Gy ≥33%, RCA V50, V55, V60 Gy (Gr≥3 CEs); LAD V20 Gy ≥9%, LAD mean, LAD V15 Gy, LAD V25 Gy, RCA V50, V55, V60 Gy (arrhythmia); LAD V50 Gy (PCE), LAD V30 (mortality); Various LAD and RCA variables linked with any CE, arrhythmia, PCE, mortality |
| Miller 2024^19^ | Esoph. | Retrospective; 238 | 1.9y | 2007-2019; 35% 3D-CRT, 65% IMRT; 41.4-66Gy/23-33 fx | 63y (med.), 83% M, 60% HTN, DM 27%, 11% MI, prior AFib 9%, 36% current/recent smoker, 42% statins | Afib; MACE (AFib, HF, vent. arrhythmias, sudden death) | 1y Afib 20% (med. time 4 mo); 33% MACE (63% arrhythmias, 17% HF, 9% MI, 5% PCI, 4% CV death), 1y MACE 26% (med. 11 mo) | WH, chambers; Mean, max, V5-50 Gy | LA mean, V5, V20 (Afib), LA V5, LV V5, RV V10 Gy (MACE); Increasing LA dose linked with incident Afib and worse outcomes; PVs not analyzed |
| Nguyen 2024^20^ | Esoph. | Retrospective; 122 | 1.82y | 2010-2022; 48% 3D-CRT, 52% IMRT; med. 59.4 Gy/33 fx | 95% ≥55y, 87% M, 74% any smoker, 67% EtOH, 52% HTN, 38% HLD, 17% DM, 20% BMI>30, 8% HF, 29% arrhythmia, 31% pHTN | Gr≥3 CTCAEv5 CE (pericarditis, ACS, HF, valve disease, arrhythmia) | 30% CE overall (17% Gr≥3) | Whole heart, chambers, myocardium; D5-D100 Gy and V5-V70 Gy | LA V15 Gy ≥77 cc; LA V15 Gy associated with any Gr≥3 CE; CA not evaluated, composite outcome (conduction + ischemic), limited sample size |
| Atkins 2024^21^ | Lung | Retrospective; 748 | 5.5y | 1998-2014; 78% 3D-CRT 78.1%, 22% IMRT, med. 64Gy | 65y (med.), 51% M, 92% any tobacco, 50% HTN, 48% HLD, 14% DM, 14% arrhythmia, 8% HF, 36% CHD; 59% ≥mod-heavy NIAAA EtOH use level | Gr≥3 CTCAEv4 arrhythmia events, grouped: 1) AFib 2) atrial flutter, 3) other (non-AFib) SVT, 4) bradycarrhythmia, 5) VT/aystole | 17% Gr≥3 arrhythmia (2y 12%, med. 2y); 2y Afib (2y 8%), Aflut (3%), other SVT (2%), bradyarrhythmia (1%), VT/asystole (1%) | Chambers, CAs (LMCA, LAD, LCx, RCA, PDA), AVN, SAN; Mean, max, V5-60 Gy (%, mL) | PV V5 Gy ≥15 cc (Afib); LCx V35 Gy ≥1.8 cc (Aflut); PV V55 Gy ≥31% (SVT) RCA V25 Gy ≥1.9 cc (bradyarrhythmia), LMCA V50 Gy ≥0.5 cc (VT/asystole); Distinct arrhythmia classes associated w/ dose to discrete sub-structures (PV dose with Afib and SVT), LCx with Aflut, RCA dose with bradyarrhythmia, and LMCA dose with VT/asystole |
| Omidi 2024^22^ | Lung | Prospective; 9 | 6 mo. | NR; VMAT; mean 57Gy (typically 2Gy/fx) | 67y (med.), 4/9 M, 8/9 any smoker, 5/9 HTN, 1/9 AFib, 3/9 DM | LGE, LS | 4% 6-mo LGE | LV myocardium | NA; greater LGE changes in LV regions receiving >50 Gy |
| Sławiński 2024^23^ | Lung | Prospective; 43 (23 cases, 20 controls) | 3 mo. | 2020-2022; IMRT; med. 66 Gy/30 fx | 65y (med.), 58% M, 61% HTN, 16% CAD, 14% HF, 33% AFib, 16% HLD, 91% any smoker | CV biomarkers: TIMP-1, MMP-9, ICAM-1, hs-TnI, BNP | Inc ICAM-1 immediately after RT (among statin users) | WH, pericardium, chambers, CA (LAD, LCx, RCA); Mean, V5, 25, 30Gy | Mean LAD, mean LCx; LAD and LCx dose correlated with ↑VCAM-1 at 3 mo. post RT |
| Sławiński 2024^24^ | Lung | Prospective; 43 (23 cases, 20 controls) | 3 mo. | 2020-2022; IMRT; med. 66 Gy/30 fx | 65y (med.), 58% M, 61% HTN, 16% CAD, 14% HF, 33% AFib, 16% HLD, 91% any smoker | RV GLS, RV FWLS, 3DRVEF, TAPSE | 40% (any) RV toxicity | WH, pericardium, chambers, CA (LAD, LCx, RCA); Mean, V5, 25, 30Gy | Mean RV; Mean RV dose correlated with 3DRVEF immediately post-RT |
| Tohidinezhad 2024^25^ | Lung | Retrospective; 374 | 3y | 2015-2022; 45-65 Gy/15-20 fx BID or 60-66 Gy/30-33 fx | 69y (med.), 57% M, 54% BMI ≥28, 18% DM, 62% HTN, 36% CHD 36%, 25% current smoker, 52% current EtOH | Afib | 11% Afib | WH, chambers, SAN, AVN; Min, mean, max, V5-60Gy (5 Gy bins) lated. The GTV of the primary tumor (GTVp) and lymph node(s) (GTVn) were also extracted. | LA Dmax; LA max associated with Afib; PVs not contoured |
| Walls 2024^26^ | Lung | Retrospective; 478 | 1.8y | 2015-2020; 29% 3D-CRT, 71% IMRT; 55 Gy/20 fx | 70y (med.), 53% M, 51% HTN, HLD 57%, 96% any smoker, 23% ACS, 11% arrhythmia, 9% HF, 13% CVA | CE: ACS, acute HF, Afib, | 6% Afib, 7% HF, 3% ACS (med. 16 mo) | Heart base (RA -including the SAN-, SVC, aortic root, LMCA, proximal LAD and RCA); Dmax, VX Gy (5Gy bins) | Heart base Dmax; was associated with ↑pooled CE |
| Walls 2024^27^ | Lung | Retrospective; 420 | 1.8y | 2015-2020; 30% 3D-CRT, 70% IMRT; 55 Gy/20 fx | 70y (med.), 52% M, 12% EtOH units/wk >7, 51% HTN, 60% HLD, 21% DM, PY 40 (med.), 25% CAD, 2% non-Afib arrhythmia 2%, 5% HF, 11% CVA | CTCAE v5 AFib | 6% Afib | PV, SAN, LA; Mean, max, V10, V55 Gy, SAN max. LA V20 | LPV V55 Gy, RPV V50 Gy; both were associated with Afib (SAN Dmax and LA V20 Gy were NS) |
| Yoo 2024^28^ | Lung | Retrospective; 508 | 3.01y | 2008-2019; 34% 3D-CRT, 66% IMRT; 60-66 Gy/30-33 fx | 67y (med.), 81% M, 42% HTN, 23% DM, 3% AFib, 8% CAD, 80% CAC>0 (med. 109), 77% any | Afib | 5% Afib | WH, chambers, LAD, LCx, RCA, SAN, AVN; Mean, max, V5-60Gy (5 Gy bins) | SAN Dmax; was the key predictor of Afib in ML model (AUC of >0.8 for institutional and ext. cohorts); PVs not analyzed |
| Yegya-Raman 2024^29^ | Lung | Retrospective; 335 | 3.3y | 2017-2021; 6% 3D-CRT, 59% IMRT, 35% PBT; 60-70 Gy/30-35 fx | 68y (med.), 46% M, PY 38 (med.), 64% HTN, 48% HLD, 21% DM, 41% CHD, 10% HF, 9% CVA, 15% Afib, 55% statins | MACE (ACS, CA revasc., CV death, HF) | 2y MACE 10% | WH, LAD, LV; Mean, max, Dx%(Gy) from 5-95% | None (for MACE); No CS parameter associated w/ MACE; LAD V15Gy links w/ mortality; low CV event rate, low LAD V15 Gy exposure (med. 1%) |
| Butler 2024^30^ | Mixed | Retrospective; 539 (230 lung, 174 breast, 119 HL, 16 esophageal) | 4.9y | 2004-2022; Technique (NR); NSCLC (66 Gy/30 fx), breast (50 Gy/25 fx), HL (30 Gy/20 fx), esoph (50 Gy/25fr) | 58y (med.), 40% M, 49% former/current smoker, 35% HTN, 14% DM, 26% statin, 16% prior Gr≥3 CE, 4% AFib | CTCAE Gr≥3 AFib | 5y Afib 5% (11% NSCLC; 8% esoph, 1% breast/HL | PVs (L/R, S/I), LA, SAN, LMC, LAD, LCx; Mean, max | PV Dmax ≥40 Gy; had a higher risk of Afib (4% v 1% when MARFS 0-1; 12% v 0% when MARFS 3+); higher max for all individual PVs and combined PV associated w/ Afib |
| Choi 2023^31^ | Breast | Retrospective; 84 (29 case, 55 control) | 6.6y | 2005-2015; 3D-CRT; 50.4 Gy/28 fx | 48% ≥55y, 2% smoking, 36% HTN, 13% DM, 2% CHD | ACE | NA | LV, LAD, RCA; NA | Mean, Dmax: LV, LAD, RCA; LV, LAD, RCA dose model had an AUC 0.94/0.83 (for ACE) in cross-validation/independent testing. |
| Holm Milo 2023^32^ | Breast | Retrospective; 612 | 7.3y | 2005-2016; 3D-CRT; 55% 50 Gy/25 fx, 28% 40 Gy/15fx | Cases: 66y (med.), 29% CHD, 22% HTN, 20% HLD, 8% DM | CAD | 1% CAD (med. 3.8y) | WH, chambers, LAD, LCx, RCA, PDA; Mean, D0.1 cm3, V5Gy | LAD; CAD was apt to occur in highest L-minus-R dose area (noted at distal LAD), where highest L v R event ratio was observed; low CAD incidence |
| Lai 2023^33^ | Breast | Retrospective; 2,158 | 7.9y | 2005-2017; 96% 3D-CRT, 4% IMRT; typically 50.4 Gy/28 | 51y (med.), 8% BMI ≥30, 4% CHD, 9% DM, 25% HTN, 10% HLD | major ischemic event | 5-, 10-, 15-y rates: 2%, 5%, 8% | WH, chambers, LAD, LCx, RCA; Mean, max, V5-60 Gy (5 Gy bins) | LV V25>4% for left sided; LV V25 >4% model had best goodness of fit and discrimination in L-sided |
| Teimouri 2023^34^ | Breast | Retrospective; 30 | 3 mo. | 2019-2020; 3D-CRT; 50 Gy/25 fx | 47y (mean), BMI 22 (mean) | ECG and ECHO changes | T-wave inversion 47% | WH, chambers; LV V5 Gy | LV V5 Gy; associated with shortened ST segment and ↑ LVESD; small sample size |
| Toma 2023^35^ | Breast | Retrospective; 68 | 1.8y | 2015-2021; 46% 2D, 44% 3D-CRT, 10% IMRT | 55y (mean), 71% post-menopause, 41% CVD, 78% DM | ECG and ECHO changes | NR | Chambers, LAD, RCA, LCx; Mean, max, V25, V30 Gy | LAD Dmax, mean; LAD Dmax >47Gy and mean >33Gy associated with >5% LVEF decline |
| Wang 2023^36^ | Breast | Prospective cohort; 61 | 1y | 2013-2015; IMRT; 45-50.4 Gy/25-28 fx or 40-42.5 Gy/15-16 fx | 50y (med.), 11% BMI ≥30, 10% smoking, 15% HTN, 34% HLD, 10% DM | SPECT perfusion defect | NR | LV (20 segment AHA model); Mean | LV segment mean; Pts with new perfusion defects had higher RT dose in LV segment 3 (vs no defects) |
| Cai 2023^37^ | Esoph. | Retrospective; 716 | 7.8y | 2010-2016; 21% 3D-CRT, 79% IMRT 78.9%; med. 60 Gy | 65y (med.), 75% M, 49% PY ≥20, 26% HTN, 12% DM, 29% HLD, 18% CHD | Gr≥3 CTCAE, ACS/CHF | 3y CIF: total gr≥3 12% (med. 14 mo), ACS/CHF 5%, arrhythmia 5% | WH, chambers, LAD, LCx, RCA, pericardium; Mean, max, V5-60 Gy (V5Gy bins) | LAD, LCx; CS equal to WH for overall Gr≥3 CE; LAD/LCX > WH for ACS/CHF |
| Umezama 2023^38^ | Esoph. | Prospective cohort; 23 | 6.8y | 2013-2015; 3D-CRT; typically 60 Gy/30 fx | 64y (med.), 57% M, 70% smoking, 30% HTN | Gr≥3 CE (ACS, arrhythmia, HF, pericarditis, VHD, cardiomyopathy) | 5y gr≥3 15% | WH, LMCA, LAD, LCx, RCA, LV (excluding blood pool); LV: V5-V60Gy; CA: mean | LV V45Gy ≥2%; was the best predictor for RT-induced myocardial damage; small sample size |
| van der Ree 2023^39^ | Esoph. | Retrospective analysis of prospective cohort; 20 | NR | NR; Technique (NR); 41.4 Gy/23 fx | 64y (mean); 80% M, LVEF 57% (med.) | GLS, segment strain | Mild strain worsening; no change in LVEF | LV (17-segments); >20, 25, 30 Gy | LV segments >30Gy; Segments >30Gy experienced mild strain worsening; unchanged LVEF |
| Ahmadsei 2023^40^ | Lung | Retrospective; 60 | 2.2y | 2014-2021; IMRT; med. 45 Gy/8 fx | 68y (med.), 75% M, 22% CAD, 2% IHD, 5% MI, 17% nIHD, 2% pHTN, 15% VHD, 7% CVA, 15% Afib, 17% other arrhythmia, 50% HTN, 18% HLD, 17% DM, 80% smoking hx | Gr≥3 CTCAEv5 CE | 20% CE (med. 1.8y) | WH, chambers, GV, valves, CA, SAN. AVN; Mean, max, D0.1, D0.5, D1cc | None; No cardiac DVH parameters associated w/ CE; small sample size/limited events. |
| No 2023^41^ | Lung | Retrospective; 233 | 6.1y | 2006-2018; >99% IMRT; dose (NR) | 68y (med.), 39% M, 13% smoker, 22% DM, 52% HTN | Gr≥3 CE (myocardial, constrictive, valvular, and conduction) | 22% CE (med. 22 mo) | LMCA, LAD, LCx, TotalLeft; V15 Gy | TotalLeft V15Gy; V15 thresholds: 1.5 cc (LAD), 0.2 cc (LMCA), 0.7 cc (LCx), 2.5 cc (TotalLeft), 37 cc (LV); TotalLeft V15 Gy and CAC >5 associated with CE; similar to findings from Atkins 2021; CA performed >LV |
| Tjong 2023^42^ | Lung | Retrospective; 500 (dev.), 201 (internal validation), 102 (ext. validation) | 2.0y | 2005-2021; 20% 3D-CRT, 80% IMRT; med. 60 Gy/30 fx (validation) | 71y (med.), 45% M, 77% smoker, 65% HTN, 29% DM, 8% CHF, 31% CHD | MACE (UA, HF, MI, CA revasc., CV death) | 4y MACE in low vs high-risk groups: 6% vs 21% | LAD; V15Gy | LAD V15Gy; CHyLL model externally validated and better than MHD in predicting MACE |
| Bates 2023^43^ | Peds | Retrospective; 25,481 (n=12,288 RT) | >25y | 1970-1999; 97% 2D or 3D-CRT | NA - peds; 33% smoking hx in adulthood | CAD, HF, VHD, arrhythmia | 35y CIF: 4% CAD, 4% HF, 1% arrhythmia, 1% VHD | WH, chambers, CAs, valves; Mean | Mean 5-9.9 Gy: RCA, LV (CAD); TV, RV (VHD); Mean 5-9.9Gy to RCA, LV, valves ↑ CE risk |
| Errahmani 2022^44^ | Breast | Case-control; 116 (21 cases and 95 controls) | 7y | 2009-2013; 3D-CRT; typically 50 Gy/25 fx | 66y (med.), 62% HTN, 5% DM | Arrhythmia | NA | WH, chambers; Mean | None; No statistically significant results; possible trend with RA dose and R-sided BC with arrhythmia |
| Van velzen 2022^45^ | Breast | Retrospective; 5,300 | 4.4y | 2005-2016; 3D-CRT; dose (NR) | 60y (mean), 35% CAC>0 | HD | 2% HD | Chambers, LAD, LCx, RCA, PA, aorta; Mean | Mean LA, RV, LV, LAD, LCx, RCA, PA, aorta; Increasing RT dose to all CS (except RA) ↑ HD risk; no baseline CV risk assessment |
| Zureick 2022^46^ | Breast | Retrospective; 375 | 4y | 2012-2018; 98% 3D-CRT, 2% IMRT; typically 50-50.4 Gy/25-28 fx or 42.6 Gy/ 16 fx | 60y (med.), 37% BMI >30, 43% smoking | MCE (UA, HF, MI, CA revasc., CV death); any CE (major CE + VHD, arrhythmia, pericarditis) | 10% CE, 6% MCE | WH, LAD; Mean, max (EQD2) | LAD mean ≥3 Gy and max ≥7 Gy (MCE); were associated with MCE; did not evaluate volumetric predictors |
| Locquet 2022^47^ | Breast | Prospective; 186 | 6 mo. | 2017-2022; 60% 3D-CRT, 39% IMRT; typically 40.05-42.56/15-16 fx | 58y (mean), BMI 26 (mean), 22% HTN, 4% DM, 11% on statins, 31% former and 16% current smoker | Subclinical LV dysfunction (GLS reduction >15%) | 14% subclinical LV dysfunction | WH, LV; mean, min, max, V5, V20 Gy | LV V5 Gy; LV V5 Gy associated with subclinical LV dysfunction at 6 mo by GLS reduction >15% |
| Wang 2022^48^ | Esoph. | Retrospective; 355 | 5.6y | 2005-2015; 66% IMRT, 34% PBT; 50.4 Gy/28 fx | 62y (med.), 90% M, 67% smoking, 42% BMI ≥30, 16% CAD, 56% HLD, 61% HTN, 25% DM | MCE (MI, CA revasc., IHD death) | 4% MCE (med. 16 mo) | WH, chambers, LMCA, LAD, LCx, RCA; Mean, V5, V30, V50 Gy | LAD V30Gy ≥10%; mean LMCA associated with mortality; mutual promotion effect of HLD and RT on MCE |
| Nabialek-Trojanowska 2022^49^ | HL | Retrospective; 12 | 4.3y | 2008-2018; 50% 2D/3D RT; 50% IMRT; med. 33 Gy/18 fx | Ages 20-65y; others NR | LV longitudinal strain | NR | LV, LAD; Mean, med. | LV, LAD; LAD med. dose associated with LV GLS, LAD Dmax with anterior LS |
| Cho 2022^50^ | Lung | Retrospective; 133 | 3.8y | 2008-2015; 83% 3D-CRT, 14% IMRT; 60-66Gy/30 fx | 67y (med.), 86% M, 29% current smoker, 17% DM, 32% HTN, 6% CHD | Gr≥2 cardiac CTCAEv5 | 32% CE | WH, LVmyo; Mean, V5, V30, V50 Gy | None; On MVA, maximum LV SUV and MHD associated w/ CE (only MHD selected in MVA) |
| Kim 2022^51^ | Lung | Retrospective; 321 (321 NSCLC; 239 SCLC) | 2.7y | 2008-2019; 35-49% 3DCRT, 52-66% IMRT; 60-63Gy/30-35 fx | NSCLC: 67y (med.), 42% HTN, 23% DM, 3% Afib, 8% CAD | Afib | 5% Afib | WH, chambers, LAD, LCx, RCA, SAN, AVN; Mean, max, V5-V60 Gy in 5Gy bins | Dmax SAN, RA, LA; SAN Dmax associated with Afib and mortality; PVs not evaluated |
| Tjong 2022^52^ | Lung | Retrospective; 701 | 2.0y | 2003-2014; Development: 16% IMRT, 84% 3D-CRT; med. 63 Gy/33 fx | 65y (med.), 51% M, 92% any smoker, 51% HTN, 49% HLD, 14% DM, 14% arrhythmia, 6% VHD, 35% CHD | MACE (UA, HF, MI, CA revasc., CV death) | CHyLL model predicted low vs high risk of 4y MACE: 7% vs 24% | LAD; V15Gy | LAD V15Gy; CHyLL estimated LADV15 constraints based on CV risk: for CHD(-) LAD V15 <11%, for CHD(+) <28% to remain low-risk |
| Tagami 2021^53^ | Breast | Retrospective; 94 | 3.6y (L), 2.9y (R) | 2006-2019; NR | 69y (med.), BMI 30 (med.), 53% HLD, 63% HTN, 10% smoker | CAD (by CCTA) | L vs R: 86% vs 46% (all CCTA+) | WH, LAD, LCx, RCA; Mean | MHD, mean LAD; Both strongly correlated with CAD (↑ of 21%/Gy mean LAD dose, 95%/Gy MHD); imaging endpoint |
| Trivedi 2021^54^ | Breast | Retrospective; 61 | 1y | 2009-2017; 3D-CRT; 50 Gy/25 fx or 42.4 Gy/16 fx | 60y (mean), BMI 27 (mean), 30% smoking, 20% DM, 46% HTN, 49% HLD | Strain | NR | Basal, mid, and apical regions for the 6 LV walls; Mean | Mean LV segment; Segmental LV dose associated with strain worsening at 6 wks, present at 12 mo |
| Van den Bogaard 2021^55^ | Breast | Retrospective; 163 | 9.2y | 2005-2008; 3D-CRT; 50.4 Gy/28 fx | 59y (med.), 7% BMI ≥30, 30% HTN, 16% HLD, 13% DM, 6% CHD | ACE | 4% ACE; 9y 11% vs 3% (plaque vs non-plaque) | WH, LV, LAD, LAD plaque; Mean, V5Gy | Mean LAD plaque, LV V5Gy; Mean LAD plaque dose strongest predictor for ACE in plaque(+); LV-V5Gy associated with ACE in plaque(-); no cut-point analysis |
| van Aken 2021^56^ | H&N | Retrospective analysis of prospective cohort; 750 | 3.4y | 2007-2016; 12% 3D-CRT, 88% IMRT; dose (NR) | 39% >65y, 75% M, 88% any smoking, 12% DM, 31% HTN, 8% arrhythmia, 5% HF | ischemic cerebrovascular event (ICVE) | 5r ICVD 5% | Carotid artery; Dmax, mean, V10 Gy bins | V10Gy (cc); Carotid V10Gy associated with risk of ICVE; no cut-point analysis |
| Atkins 2021^57^ | Lung | Retrospective; 701 | 2.0y | 2003-2014; 77% 3D-CRT, 23% IMRT; med. 66 Gy/33 fx | 65y (med.), 51% M, 92% any smoker, 52% HTN, 49% HLD, 14% DM, 14% arrhythmia, 6% VHD, 35% CHD | MACE (UA, HF, MI, CA revasc., CV death) | 10% MACE (4% 1y; med. 20 mo) | WH, chambers, total CA, LMCA, LAD, LCx, RCA, PDA; Mean, max, V5-V60 Gy (5 Gy bins) | MACE: LAD V15Gy ≥10%, LCx V15Gy ≥14%, LV V15Gy ≥1%, total coronary mean ≥7 Gy; ACM: LAD V15Gy ≥10%; Pre-existing CHD might alter choice CS constraints; CS dose and CHD interaction; high 3D-CRT use, pre-ICI era; no PV/conduction contours |
| Atkins 2021^58^ | Lung | Retrospective; 701 | 1.7y | 2003-2014; 75-87% 3D-CRT, 13-25% IMRT; 60-66Gy/30-33 fx | 65y (med.), 51% M, 92% any smoker, 52% HTN, 49% HLD, 14% DM, 14% arrhythmia, 6% VHD, 35% CHD | MACE (UA, HF, MI, CA revasc., CV death) | 10% MACE (4% 1y; med. 20 mo) | WH, LAD; V15Gy | LAD V15 Gy; Prediction of LAD V15Gy using MHD is insufficient; when LAD V15 is high (>10%), and MHD low (<10 Gy), MACE risk remains high |
| Xu 2021^59^ | Lung | Retrospective analysis of prospective cohort; 225 | 2.2y | 2009-2014; 61% IMRT, 39% PSPT; 60-74Gy/30-37 fx | 66y (med.), 53% M, 92% any smoker, 19% CHD | CAE (Gr≥3 CTCAE arrhythmia, ACS, pericarditis, cardiomyopathy,  ventricular dysfunction, cardiac arrest, and  CHF); ∆hs-cTnT peak | 11% Gr≥3 CAE (med. 9 mo) | WH, pericardium, chambers; Mean, max, V5-V70 Gy (5 Gy bins) | LV V35, V15Gy; with strongest correlation coefficients for ∆hs-cTnT; only MHD accounted for in multivariable model for CAE |
| Pao 2020^60^ | Esoph. | Retrospective; 126 | 1.2y | 2008-2018; IMRT; 66-66.6 Gy/33-37 fx | 57y (med.), 96% M, 91% any smoker, 20% HTN, 12% DM, 6% CVD | PCE (Gr≥2) | Gr≥3 PCE 6% (med. 7 mo) | WH, pericardiun; V5, V10-V60 (10 Gy bins) | Pericardium V30Gy > 66%, V40 Gy > 55%, V60 Gy >25%; associated with grade ≥3 PCE |
| Takeuchi 2020^61^ | Esoph. | Retrospective; 83 | 4.8y | 2001-2016; 3D-CRT; 60-66 Gy/30-33 fx | 55% ≥69y, 85% M, 11% DM, 87% any smoker, 15% CVD | PCE | Symptomatic PCE 14% | WH, pericardium; Mean, V5-V100 Gy | Pericardium V80Gy; and MHD associated with symptomatic PCE |
| Umezawa 2020^62^ | Esoph. | Prospective; 19 | 1.5y | 2013-2015; 3D-CRT; med. 60Gy/30 fx | Mean 62y, 12/19 any smoker, 4/19 HTN, 6/19 HLD, 11/19 CAC | LGE | NA | WH, LV, LMCA, LAD, LCx; V10-V60 (10 Gy bins) for LV and WH, mean for CAs | LV myocardium; 13/19 patients with dose >30 Gy showed LGE |
| Jang 2020^63^ | Lung | Retrospective; 258 | 2.3y | 2008-2018; 3D-CRT/IMRT; 50-72 Gy/25-40 fx | ASCVD high risk: 66y (med.), BMI 23 (med.), 95% M, 75% current smoker, 7% CVD | MACE (UA, HF, MI, CA revasc., and CV death); ACE; Gr≥3 cardiac CTCAE | 4% MACE; 11% Gr≥3 CTCAE | WH, chambers; Mean, V5, V10-60 Gy (10 Gy bins) | LV V60Gy>0; associated with ACS in intermediate-high ASCVD risk >7.5% (but not low risk) |
| Niedzielski 2020^64^ | Lung | Retrospective analysis of prospective cohort; 141 | 2.4y | NR; IMRT or PBT; ≥60 Gy/30 fx | 64y (mean), 55% M, 89% HTN, 13% DM, 67% HLD | Gr≥2 PCE | 50% Gr≥2 PCE | WH, chambers; Mean, max, V5-V70Gy | LA mean, V5, V20, V25, V30, V35, V55, V60, V65; RV Dmax; Models with WH and LA, RV dose had highest performance; high performance (AUC = 0.88) in final fitted elastic net model |
| Abouegylah 2019^65^ | Breast | Retrospective; 202 | 6.7y (L), 7y (R) | 2000-2014; 3D-CRT; dose (NR) | L v R: 47y v 51y (med.), 5% v 7% (CHD), 20% v 30% (HTN) | Arrhythmia, ischemia, LVEF | L v R: Arrhythmia (14% v 1%), ischemia (9% v 1%), EF ↓ >10% (22% v 24%; ns), EF ↓ >15% (8% v 6%; ns) | LAD, chambers; Mean, max, med., V10, V20, EUD | RV Dmax, RV EUD, LV V10Gy, LV EUD, LAD Dmax, LAD EUD; LV, RV, LAD EUD correlated with ↓LVEF >10%; baseline CV risk not accounted for |
| Van den Bogaard 2019^66^ | Breast | Cross-sectional; 109 | 7y | 2005-2011; 3D-CRT; 50.4 Gy/28 fx | 55y (med.), 6% DM, 17% HTN, 6% HLD, 28% smoking, 1% CHD | TTE changes | Abnormal LVEF 14% | LV, LMCA, LAD, LCx, RCA; Mean, max, V5Gy bins | LMCA Dmax; associated with LV GLS (but not LVEF or DD) |
| Walker 2019^67^ | Breast | Prospective cohort; 79 | 6 m | 2015-2017; 3D-CRT; 50 Gy/25 fx | 58y (mean), BMI 24 (mean), 47% smoking, 24% HTN, 6% DM, 30% HLD | Subclinical LV dysfunction (GLS reduction >10%) | 6% mean ↓ GLS | WH, LV; Mean, D2Gy, V20Gy | LV V20Gy >15%; associated with subclinical LV dysfunction |
| Wennstig 2019^68^ | Breast | Retrospective; 182 | 5y | 1992-2012; 3D-CRT; 50 Gy/25 fx | 64y (med.) | Coronary intervention | NA | WH, LMCA, LAD, LCx, RCA; Mean, max, med. | Mean LAD; Mean 5-20 Gy and >20 Gy to the mid-LAD associated with coronary intervention; older RT era/higher cardiac dose |
| Trivedi 2019^69^ | Breast | Prospective; 40 | 12 mo. | 2009-2012; 3D-CRT; 50 Gy/25 fx or 42.4 Gy/16 fx | 60y (med.), BMI (mean) 29, 43% HTN, 53% HLD, 20% DM, 33% smoking | LVEF, GLS | 40% GLS decline >10% | WH, LV; Mean | None; No dose associated with LVEF or GLS; small sample size. |
| Borkenhagen 2019^70^ | Lung | Retrospective; 76 | 1.2y | 2010-2015; NR; med. 60 Gy/30 fx | 64y (med.), 55% F, 96% any smoker, 37% CHD, 22% DM | Arrhythmia, PCE, VHD | Arrhythmia 7%, PCE 17%, VHD 1% | Chambers, pericardium, heart base; Mean, max, V30, V45Gy | Ventricles V45Gy; predicted CE; no coronary or PV/conduction contours |
| Hotca 2019^71^ | Lung | Retrospective; 155 | 1.6y | 2004-2014; IMRT; med. 64 Gy/32 fx | 66y (med.), 29% CHD, 17% DM, 35% HLD, 48% HTN, 90% any smoker | ECG abnormalities (arrhythmic, ischemic/pericardial, or non-specific) | Rate of arrhythmic, pericardial/ischemic, non-specific ECG changes (66%, 35%, 67%) | WH, chambers, IVC, SVC, aorta, lung, PA; Mean, max, min dose to the hottest x% V, Dx%[Gy] (x=5–95 in 5% steps) | SVC min; Dose to SVC associated with non-specific ECG changes; no PV/conduction system contours |
| Xue 2019^72^ | Lung | Retrospective; 94 | 4.8y | NR; mostly 3D; 60-85.5 Gy/2-3.8 Gy fx | 54% >65y, 17% F, 89% smoker | PCE | PCE 40% | WH, pericardium, mean, V5, 30, 55 Gy | Pericardium mean, V55 Gy; predicted PCE |
| Milgrom 2019^73^ | Mixed | Retrospective; 20 | 2.66y | 2006-2017; 70% 3D-CRT, 25% IMRT, 5% PBT; med. 60 Gy/30 fx | 53y (med.), 70% F, 50% BMI ≥30, 60% HTN, 55% HLD, 25% DM | CAC score | NR | WH, LMCA, LAD, LCx, RCA; Mean, max, V10-50 Gy (10 Gy bins) | LAD Dmax, V5 Gy, V40 Gy; LCX V5 Gy; RCA Dmax, mean, V40 Gy; Dose exposure to CA strongly correlated with subsequent segmental CAC score (but not MHD) |
| Mansouri 2019^74^ | Peds | Case-control; 239 | 19.7y | 1945-2000; NR | NA (peds) | HF | NA | WH, LV; Mean, V20, V30, V40 Gy | LV V30 Gy; associated w/ HF risk; phantom anatomy reconstruction; no cut-point analysis |
| Jagsi 2018^75^ | Breast | RCT; 54 | 1y | 2006-2012; 3D-CRT arm: 50-50.4 Gy/25-28 fx; IMRT arm: 52.2 Gy/30 fx | 50y (mean), BMI 28 (mean), 4% CHD, 19% HTN, 13% HLD, 37% smoker | SPECT LAD perfusion, LVEF | NA | LV, LAD; V5Gy | None; No CS associated with LVEF; 5% drop in LVEF with 3D-CRT vs IMRT w/ DIBH |
| Reshko 2018^76^ | Lung | Retrospective; 75 | 2.2y | 2007-2017; SBRT; 48 Gy/4 fx | 70y (mean), 51% F, 57% 'cardiac history', 99% any smoking, 26% DM, 86% HTN | CE (arrhythmia, CHF, CAD, VHD) | 25% CE (mean 19 mo) | Chambers, LMCA, LAD, LCx, RCA, valves, AVN; Mean, D0.03 cc | None; No significant association observed |
| Yegya-Raman 2018^77^ | Lung | Retrospective; 140 | 3.95y | 2007-2015; IMRT; 60-66 Gy/30-33 fx | 64y (med.) | supraventricular arrhythmia, ACS/CHF | 28% symptomatic CE (med. 15 mo) | WH, chambers, LAD; Mean, V5, V30, V50 Gy | Mean LV, RV, LAD; Dose to LAD and ventricles associated with ACS/CHF; small sample size |
| Van den Bogaard 2017^78^ | Breast | Retrospective; 910 | 7.6y | 2005-2008; 3D-CRT; 50.4 Gy/28 fx | 59y (med.), 58% ≥1 ACE RFs, 4% CHD, 3% VHD, 31% HTN, 7% DM | ACE | ACE: 5y 2%, 9y 4% | WH, chambers; Mean, max, V5Gy bins | LV V5 Gy; MHD; LV V5 Gy most associated with ACE (>MHD); ACE ↑ by 17% per Gy; best NTCP model consisted of LV V5Gy, age, weighted ACE risk score; CA not analyzed |
| Ogino 2017^79^ | Esoph. | Retrospective; 86 | 4.7y | 2000-2013; 3D-CRT; 59.4 Gy/33 fx | 68y (med.), 88% M, 43% HTN, 12% DM | Asymptomatic PCE, symptomatic PCE | 5y asymptomatic PCE 45% (symptomatic 6%) | WH, pericardium; Pericardium V5-V65 Gy | Pericardium V50, D10; Pericardium V50 ≤17% associated with fewer symptomatic PCE |
| Hahn 2017^80^ | HL | Retrospective; 175 | 10.4y | 1988-2004; 2D; 35 Gy/20 fx | 34y at dx (mean), 39% F, 5% HTN, 6% HLD, 1% DM, 35% smoking | 1) All CE (IHD, pericardial disease, conduction disorders, VHD, ventricular dysfunction, and cardiac surgeries and procedures, 2) IHD | 5y 7%, 10y 20%, 15y 37% | LAD, LCx, RCA; Mean, max, min, dose homogeneity, V5, V10, V20, V30 Gy | LAD V5 Gy, LCX V20 Gy; 1) All CE endpoint: WH and CA models were similar; 2) IHD endpoint: LCx V20 Gy, LAD V5 Gy > WH |
| Vivekanandan 2017^81^ | Lung | Retrospective analysis of prospective cohort; 78 | 6 mo. | NR; NR; 63-73Gy/30 fx | 66y (med.), 26% F, 49% normal ECG | ECG changes at 6 mo | 38% ECG changes at 6-mo | WH, pericardium, AVN, chamber walls; Mean, VXGy (1Gy bins) 1-Gy dose-bins | None; Heart-PC6, Pericardium-PC5, and LA-Wall-PC6 scores did not associate with ECG changes |
| Wang 2017^82^ | Lung | Retrospective analysis of prospective cohort; 112 | 8.8y | 1996-2009; 3D-CRT; 70-90 Gy | 58y (med.), 67% F, 22% WHO/ISH 10-yr risk ≥20% | Symptomatic CE (arrhythmic, ischemic, pericardial) | 23% CE (med. 26 mo) | WH, LV; Mean, V5, V30 Gy | LV V5 Gy; associated with CE, accounting for baseline CV risk; small sample size, limited CS analysis |
| Wang 2017^83^ | Lung | Retrospective analysis of prospective cohort; 112 | 8.8y | 1996-2009; 3D-CRT; 70 - 90 Gy | 58y (med.), 67% F, 22% WHO/ISH 10-yr risk ≥20% | Symptomatic CE (arrhythmic, ischemic, pericardial) | 23% CE overall | WH, chambers, LAD; Mean, V5, V30, V60 Gy | LA, RA, LV V30Gy; Arrhythmia (LA, RA), ischemic events (LAD, LV), pericardial events (RA, LA); small sample size, no multivariable model for CE |
| Cao 2016^84^ | Breast | Retrospective; 137 | 2.2y | 2009-2011; IMRT (50 Gy/25 fx) | 45-48y (med.), BMI 22-23 (med.); 16% CHD/HTN/DM | LVEF | LVEF 8% (Trastuzumab+RT); 4% (RT alone) | WH, LV; Mean, D1-D95, V1-V50 Gy | LV D30-D45, D65-D75, V6-V15 Gy; Mean and low LV dose associated with ↑ acute LVEF dysfunction; concurrent Trastuzumab |
| Cao 2015^85^ | Breast | Retrospective; 143 | 6 mo. | 2011-2012; IMRT (50 Gy/25 fx) | 44-48y (med.), BMI 23 (med.), 18-19% CHD/HTN/DM | LVDD | LVDD 35% (Trastuzumab+RT); 20% (RT only) | LV; D1-D95, V1-V50 Gy | LV Dmean, D15-D40, D60-D70, V3-V10; LV dose variables ↑ in LVDD; concurrent Trastuzumab |
| Skyttä 2015^86^ | Breast | Prospective; 58 | NA | 2011-2013; 3D-CRT; 50 Gy/25 fx or 42.56 Gy/16 fx | 63y (mean), BMI 27 (mean), 34% HTN, 2% CAD, 7% DM, 14% current smoking | hscTnT >30% | 21% with ↑hscTNT | WH, LV, LAD; NR | LV, LAD; pts with ↑hscTNT post-RT had higher WH, LV, and LAD V15 and V20 Gy |
| Moignier 2015^87^ | HL | Case-control; 33 (12 cases, 21 controls) | NR | NR; 2D or 3D-CRT; NR | Cases v controls: 30 v 36y (med); 42% v 52% M | CA stenosis (CCTA) | NA | WH, aorta, LMCA, LAD, LCx, RCA; Mean, D2% | CA segment med.; Median CA segment dose associated w/ ↑ CA stenosis |
| Cutter 2015^88^ | Peds/HL | Case-control; 289 (89 cases, 200 controls) | 18.8y | 1965-1995; 2D; various regimens | Age (NR); obesity, smoking, HTN, DM, HLD (included in RR models) | VHD | 32% VD (5% aortic, 14% tricuspid) | Valves; Mean | Valve dose >30Gy; (and esp. >40 Gy) ↑ VHD; CIF VHD lower in modern mediastinal RT |
| de Ville de Goyet 2015^89^ | Peds/HL | Retrospective; 81 (n=11 RT) | 2y | 2009-2013; TBI; 12 Gy | NA-peds | LVSD, left atrial volume (cMRI) | 2% LVSD | LV; Mean, max, V5-20 (5Gy bins), 30, 40, 50, 75, 95, 98 Gy (%) | LV mean; associated with ↑ LA volume; small sample size |
| Cao 2014^90^ | Breast | Retrospective; 45 | 2.1y | 2009-2011; IMRT; 50 Gy/25 fx | 44y (med.), BMI 22 (med), 16% HTN/DM | LVEF, any grade CE | 22% Gr≥1, 2% Gr≥2 | WH, LV, LAD; LV: mean, max D1-D95, V1-V50 Gy; LAD: max, mean | LV: V5-7Gy; Low LV dose associated with reversible early grade CE; concurrent Trastuzumab |
| Tamari 2014^91^ | Esoph. | Retrospective; 69 | 3.1y | 2001-2011; 3D-CRT; 60 Gy/30 fx | 59% ≥66y, 6% F, 81% smoker, 30% HTN, 13% DM, 7% CHD | PCE | Gr≥2 48%, Gr≥3 4% | Pericardium; Mean, V5-V60Gy (5 Gy bins) | Pericardium V30 ≥42%; associated with ↑ PCE; small sample size |
| Girinsky 2014^92^ | HL | Prospective cohort; 179 | 11.6y (RT); 42y (CCTA) | 2007-2012; IMRT; 36 Gy | 29y (med.) at dx, 42y (med.) at CCTA, 54% F | CCTA abnormalities | 5y 15% | CA origins; Mean | Mean CA origin; RT dose at CA origin associated with CCTA abnormalities |
| Dorth 2014^93^ | Head & Neck | Retrospective; 224 (135 IMRT) | 3y | 2000-2009; 61% IMRT; various fractionations | 56y (med.), 76% M, 70% smoking, 11% DM, 37% HTN, 10% HLD, 12% vascular disease | CAS | 4y 10% | Carotid artery, carotid artery bulb; mean, max, V50-70 (5 Gy bins) | Mean carotid dose: in IMRT group, HR for CAS was 1.4 per 10 Gy mean carotid bulb dose |
| Cella 2013^94^ | HL | Retrospective; 56 | 6y | 2002-2008; 3D-CRT; 32 Gy/20 fx | 28y (med.), 59% F, 14% smoker, 5% HTN, 2% HLD, 4% DM | Asymptomatic valvular heart defects | 32% VHD (med. 6y); 25% MV, 5% AV, 14% TV | WH, chambers; LA and LV V30 | LA V30 Gy, LV V30 Gy; The NTCP values increase as cardiac chamber V30 increases |
| Erven 2011^95^ | Breast | Prospective cohort; 30 | 2 mo. | NR; 3D-CRT; 50 Gy/25 fx | 54y (mean), 30% smoker, 20% HTN, BME 26 (mean), % HLD | Regional strain, strain rate | NR | LV (18-segments); Mean | LV segments >3Gy; was associated w/ worse strain |
| Cella 2011^96^ | HL | Retrospective; 56 | 5.9y | 2002-2008; 3D-CRT; med. 32 Gy/20 fx | 13% 46-70y, 59% F, 14% smoker, 5% HTN, 2% HLD, 4% DM | Asymptomatic VHD | 32% VHD (med. 6y); 25% MV, 5% AV, 14% TV | Chambers; Mean, max, V5-30Gy in 5Gy bins | LA V25 Gy, LV V30 Gy (MV), RV V30 Gy (TV); LA, LV, RV dose predicts asymptomatic VHD; valve dose not directly measured |
| Schytte 2010^97^ | Lung | Retrospective; 328 | 7.9y | 1995-2007; 3D-CRT; 60-66Gy/30-33 fx, 80 Gy/35-40 fx | NR; pts w/ prior cve excluded | CE (MI, CHF, PE, other) | NR | WH, LV, RV; Mean | None; LV mean ≥14.5 Gy had trend to ↑ mortality; did not account for baseline CV risk; limited CS |
| Gayed 2009^98^ | Lung | Retrospective; 44 | 1y | 2003-2006; 80% 3D-CRT, 20% IMRT | 66y (med.), 50% F, 7% DM, 20% HTN, 43% smoking hx, 28% fam hx of CAD, 4% obesity, 19% HLD | Myocardial perfusion defects | NR | LV (13-segments); Mean | LV segment mean; ChemoRT associated with anterior/septal MPI defects; higher LV dose in abnormal segments (vs normal segments) |
| Wei 2008^99^ | Esoph. | Retrospective; 101 | 8.4 mo. | 2000-2003; 3D-CRT; 50-50.4Gy/25-28 fx | 50% age >67y, 25% F, 73% smoker, 58% HTN, 6% Afib, 22% CAD | PCE | 28% overall | WH, pericardium; Mean, V5-65 Gy | Pericardium V30 Gy >46%; associated with ↑ PCE |
| Marks 2005^100^ | Breast | Prospective; 114 | 2y | 1998-2001; 3D-CRT; 46-50Gy in 1.8-2.0Gy/fx | 57y (med.), 31% HTN, 10% DM, 7% CAD, 32% obesity, 17% HLD, 41% smoker | Regional and global cardiac function by SPECT | 12% WMA; 42% new perfusion defects at 2y | LV; volume (%) into RT field (>50% Rx dose) | LV V23-25 Gy (>50% Rx dose) >5%; new perfusion defects occurred in 50-60% of pts with LV V23-25 Gy >5% |

**Abbreviations:** ACE, acute coronary event; ACM, all-cause mortality; ACS, acute coronary syndrome; Afib, atrial fibrillation; Aflut, atrial flutter; AHA, American Heart Association; ASCVD, atherosclerotic cardiovascular disease; AV, aortic valve; AVN, atrioventricular node; BC, breast cancer; BMI, body mass index; CA, coronary artery; CABG, coronary artery bypass graft; CAD, coronary artery disease; CAS, coronary artery stenosis; CCI, Charlson Comorbidity Index; CCTA, coronary computed tomography angiography; CE, cardiac event; CHD, coronary heart disease; CHF, congestive heart failure; ChemoIO, chemotherapy–immunotherapy; CHyLL, CHD, hypertension, logarithmic LAD V15, and CHD; CIF, cumulative incidence function; cMRI, cardiac magnetic resonance imaging; CS, cardiac substructures; D, dose; DD, diastolic dysfunction; DEGRO, German Society for Radiation Oncology; Dev, development; DIBH, deep inspiration breath hold; Disc., discovery; DM, diabetes mellitus; DVH, dose–volume histogram; ECG, electrocardiogram; EF, ejection fraction; EQD2, equivalent dose in 2-Gy fractions; Esoph., esophageal; esp., especially; EtOH, ethanol (alcohol); Ext., external; EUD, equivalent uniform dose; F, female; FWLS, free wall longitudinal strain; GLS, global longitudinal strain; Gr, grade; HD, heart disease; HF, heart failure; HL, Hodgkin lymphoma; HLD, hyperlipidemia; hs-cTnT, high-sensitivity cardiac Troponin T; HTN, hypertension; ICAM-1, intercellular adhesion molecule 1; ICI, immune checkpoint inhibitor; ICVE, ischemic cerebrovascular events; IHD, ischemic heart disease; IMRT, intensity-modulated radiotherapy; Int, intermediate; IVC, inferior vena cava; L, left; LA, left atrium; LAA, left atrial appendage; LAD, left anterior descending coronary artery; LAVI, left atrial volume index; LCx, left circumflex coronary artery; LGE, late gadolinium enhancement; LMCA, left main coronary artery; LS, longitudinal strain; LV, left ventricle; LVDD, left ventricular diastolic dysfunction; LVEF, left ventricular ejection fraction; LS, longitudinal strain; LVESD, left ventricular end-systolic diameter; M, male; MACE, major adverse cardiac event; MAFRS, Mayo atrial fibrillation risk score; MCE, major coronary events; Med., median; MI, myocardial infarction; Min, minimum; MPI, myocardial perfusion imaging; MV, mitral valve; NA, not applicable; NR, not reported; NS, not significant; NSCLC, non-small cell lung cancer; NTCP, normal tissue compliance probability; PA, pulmonary artery; PCA, principal component analysis; PCE, pericardial effusion; PDA, posterior descending coronary artery; PE, pulmonary embolism; Peds, pediatrics; PH, pulmonary hypertension; PSPT, passive scattering proton therapy; PV, pulmonary vein; PVD, peripheral vascular disease; Pts, patients; PY, pack-year; R, right; RA, right atrium; RAVI, right atrial volume index; RCA, right coronary artery; RCT, randomized controlled trial; Revasc., revascularization; RT, radiotherapy; RV, right ventricle; RVSD, right ventricular systolic dysfunction; Rx, prescription; SAN, sinoatrial node; SCLC, small cell lung cancer; SPECT, single-photon emission computed tomography; STE, speckle-tracking echocardiography; SVC, superior vena cava; SVT, supraventricular tachycardia; TAPSE, tricuspid annular plane systolic excursion; TIA, transient ischemic attack; TNBC, triple-negative breast cancer; Train., training; TTE, transthoracic echocardiogram; TV, tricuspid valve; UA, unstable angina; V, volume; VBA, voxel-based analysis; Valid., validation; VD, valvular disease; VHD, valvular heart disease; VT, ventricular tachyarrhythmia; WH, whole heart; WHO/ISH, World Health Organization/International Society of Hypertension; y, years.

^†^Study published in abstract and/or pre-print form only.

REFERENCES

1. Kang NK, Choi KH, Jeong JU, et al. Long-term risk of major cardiac events in breast cancer patients treated with intensity-modulated and 3-dimensional conformal radiotherapy: Secondary analysis of a randomized clinical trial. *Int J Cancer*. 2025;157:1395–1404.

2. Lamy J, Spoor DS, Langendijk JA, et al. Cardiac MRI-based subclinical cardiac dysfunction during 2 years after breast cancer irradiation: The MEDIRAD EARLY-HEART study. *Radiol Cardiothorac Imaging*. 2025;7:e240231.

3. Nesseler JP, Silos KD, Peony O, et al. Dynamic cardiac changes in low cardiovascular risk patients with triple negative breast cancer treated with chemo-immunotherapy. *CardioOncology*. 2025;11:62.

4. Guo FB, No HJ, Park NJ, et al. Risk factors of cardiotoxicity after breast cancer radiation therapy. *Adv Radiat Oncol*. 2025;10:101911.

5. Berlin E, Ko K, Ma L, et al. Cardiac effects of modern breast radiation therapy in patients receiving systemic cancer therapy. *JACC CardioOncol*. 2025;7:219–230.

6. Spoor DS, Boekel NB, Leeuwen FEV, et al. Predicting acute coronary events after breast cancer radiotherapy: Integrating baseline cardiovascular risk, systemic therapy, and cardiac radiation dose in the MEDIRAD BRACE study. *Eur J Cancer*. 2025;231:116055.

7. Populaire P, Defraene G, Liu Y, et al. Dose to the left ventricle is associated to the risk of acute cardiac toxicity in patients with esophageal cancer undergoing trimodality treatment. *Radiother Oncol*. 2025;209:111002.

8. Lee HI, Son J, Cho B, et al. Development and validation of a prediction model for cardiac events in patients with hepatocellular carcinoma undergoing stereotactic body radiation therapy. *Int J Radiat Oncol Biol Phys*. 2025;122:1272–1281.

9. Zhang SC, Kehayias CE, Guthier C, et al. Differential impact of left ventricular myocardium and left coronary radiation dose with incident heart failure and coronary events in lung cancer radiation therapy. *Int J Radiat Oncol Biol Phys*. 2025;123:792–805.

10. Atkins KM, Zhang SC, Kehayias CE, et al. Association of Pulmonary Artery Radiation with Pulmonary Hypertension after Lung Cancer Radiotherapy Accessed November 29, 2025. https://papers.ssrn.com/sol3/papers.cfm?abstract_id=5237824.

11. Nesseler JP, Oorloff M, Peony O, et al. Investigation of cardiovascular autonomic dysfunction diagnosed by exercise stress testing among patients treated with thoracic radiotherapy. *Int J Radiat Oncol Biol Phys*. 2025;123:e442.

12. Qiao EM, He J, Silos KD, et al. Baseline atrial volume indices and major adverse cardiac events following thoracic radiotherapy. *Front Cardiovasc Med*. 2025;12:1560922.

13. Guthier CV, Kehayias CE, Ciausu C, et al. Clinical validation and prospective deployment of an automated deep learning-based coronary segmentation and cardiac toxicity risk prediction system. *arXiv [physics.med-ph]*. 2025.

14. Carpenter DJ, Patel P, Niedzwiecki D, et al. Long-term risk of carotid stenosis and cerebrovascular disease after radiation therapy for head and neck cancer. *Cancer*. 2025;131:e35089.

15. Honaryar MK, Locquet M, Allodji R, et al. Cancer therapy-related cardiac dysfunction after radiation therapy for breast cancer: results from the BACCARAT cohort study. *CardioOncology*. 2024;10:54.

16. Quirk S, Atkins KM, Miller RJ, et al. The association of left anterior descending coronary artery radiation dose with major adverse cardiac events following modern breast radiotherapy. *Int J Radiat Oncol Biol Phys*. 2024;120:S102.

17. Perman M, Johansson K-A, Holmberg E, Karlsson P. Doses to the right coronary artery and the left anterior descending coronary artery and death from ischemic heart disease after breast cancer radiotherapy: a case-control study in a population-based cohort. *Acta Oncol*. 2024;63:240–247.

18. Jin Z, Sun X, Zhou C, Yang H, Zhou S. Cardiac substructures dosimetric predicts cardiac toxicity and prognosis in esophageal squamous cell cancer treated by radiotherapy. *Neoplasia*. 2024;48:100969.

19. Miller ED, Wu T, McKinley G, et al. Incident atrial fibrillation and survival outcomes in esophageal cancer following radiotherapy. *Int J Radiat Oncol Biol Phys*. 2024;118:124–136.

20. Nguyen V, Metges J-P, Morjani M, et al. Dose to cardiac substructures and cardiovascular events in esophageal cancer patients treated with definitive radiotherapy. *Radiat Oncol*. 2024;19:175.

21. Atkins KM, Zhang SC, Kehayias C, et al. Cardiac substructure radiation dose and associations with tachyarrhythmia and bradyarrhythmia after lung cancer radiotherapy. *JACC CardioOncol*. 2024;6:544–556.

22. Omidi A, Weiss E, Rosu-Bubulac M, Thomas G, Wilson JS. Quantitative analysis of radiation therapy-induced cardiac and aortic sequelae in patients with lung cancer via magnetic resonance imaging: A pilot study. *Int J Radiat Oncol Biol Phys*. 2024;119:281–291.

23. Sławiński G, Hawryszko M, Lasocka-Koriat Z, et al. Early effects of modern radiotherapy for lung cancer on endothelial damage and myocardial fibrosis: A prospective single-center study. *Int J Mol Sci*. 2024;25:6705.

24. Sławiński G, Hawryszko M, Lasocka-Koriat Z, et al. Effect of radiotherapy on the right ventricular function in lung cancer patients. *Cancers (Basel)*. 2024;16:1979.

25. Tohidinezhad F, Nürnberg L, Vaassen F, et al. Prediction of new-onset atrial fibrillation in patients with non-small cell lung cancer treated with curative-intent conventional radiotherapy. *Radiother Oncol*. 2024;201:110544.

26. Walls GM, O’Connor J, Harbinson M, et al. The Association of Incidental Radiation Dose to the Heart Base with Overall Survival and Cardiac Events after Curative-intent Radiotherapy for Non-small Cell Lung Cancer: Results from the NI-HEART Study. *Clin Oncol*. 2024;36:119–127.

27. Walls GM, McCann C, O’Connor J, et al. Pulmonary vein dose and risk of atrial fibrillation in patients with non-small cell lung cancer following definitive radiotherapy: An NI-HEART analysis. *Radiother Oncol*. 2024;192:110085.

28. Yoo SK, Kim KH, Noh JM, et al. Development of learning-based predictive models for radiation-induced atrial fibrillation in non-small cell lung cancer patients by integrating patient-specific clinical, dosimetry, and diagnostic information. *Radiother Oncol*. 2024;201:110566.

29. Yegya-Raman N, Ho Lee S, Friedes C, et al. Cardiac radiation dose is associated with inferior survival but not cardiac events in patients with locally advanced non-small cell lung cancer in the era of immune checkpoint inhibitor consolidation. *Radiother Oncol*. 2024;190:110005.

30. Butler S, No H, Guo F, et al. Predictors of atrial fibrillation after thoracic radiotherapy. *JACC CardioOncol*. 2024;6:935–945.

31. Choi BS, Yoo SK, Moon J, et al. Acute coronary event (ACE) prediction following breast radiotherapy by features extracted from 3D CT, dose, and cardiac structures. *Med Phys*. 2023;50:6409–6420.

32. Holm Milo ML, Slot Møller D, Bisballe Nyeng T, et al. Radiation dose to heart and cardiac substructures and risk of coronary artery disease in early breast cancer patients: A DBCG study based on modern radiation therapy techniques. *Radiotherapy and Oncology*. 2023;180:109453.

33. Lai T-Y, Hu Y-W, Wang T-H, et al. Association of radiation dose to cardiac substructures with major ischaemic events following breast cancer radiotherapy. *Eur Heart J*. 2023;44:4796–4807.

34. Teimouri K, Khoshgard K, Farshchian N, Rouzbahani M, Azimivaghar J. Investigation of electrocardiography and echocardiography changes after adjuvant radiation therapy of left-sided breast cancer. *J Med Imaging Radiat Sci*. 2023;54:495–502.

35. Toma RV, Anca Z, Trifănescu OG, et al. Early echocardiography and ECG changes following radiotherapy in patients with stage II-III HER2-positive breast cancer treated with anthracycline-based chemotherapy with or without trastuzumab-based therapy. *Med Sci Monit*. 2023;29:e941754.

36. Wang S-Y, Lin K-H, Wu Y-W, et al. Evaluation of the cardiac subvolume dose and myocardial perfusion in left breast cancer patients with postoperative radiotherapy: a prospective study. *Sci Rep*. 2023;13:10578.

37. Cai G, Li C, Li J, et al. Cardiac Substructures Dosimetric Predictors for Cardiac Toxicity After Definitive Radiotherapy in Esophageal Cancer. *Int J Radiat Oncol Biol Phys*. 2023;115:366–381.

38. Umezawa R, Ota H, Takagi H, et al. Clinical impact of radiation-induced myocardial damage detected by cardiac magnetic resonance imaging and dose-volume histogram parameters of the left ventricle as prognostic factors of cardiac events after chemoradiotherapy for esophageal cancer. *J Radiat Res*. 2023;64:702–710.

39. van der Ree MH, de Bruin-Bon RHA, Balgobind BV, et al. Dose-dependent cardiac effects of collateral cardiac irradiation: Echocardiographic strain analysis in patients treated for extracardiac malignancies. *Heart Rhythm*. 2023;20:149–151.

40. Ahmadsei M, Thaler K, Gasser E, et al. Dosimetric analysis of 17 cardiac Sub-structures, Toxicity, and survival in ultra central lung tumor patients treated with SBRT. *Clin Transl Radiat Oncol*. 2023;43:100675.

41. No HJ, Guo FB, Park NJ-I, et al. Predicting adverse cardiac events after radiotherapy for locally advanced non-small cell lung cancer. *JACC CardioOncol*. 2023;5:775–787.

42. Tjong MC, Zhang SC, Gasho JO, et al. External validation of Cardiac disease, Hypertension, and Logarithmic Left anterior descending coronary artery radiation dose (CHyLL) for predicting major adverse cardiac events after lung cancer radiotherapy. *Clin Transl Radiat Oncol*. 2023;42:100660.

43. Bates JE, Shrestha S, Liu Q, et al. Cardiac Substructure Radiation Dose and Risk of Late Cardiac Disease in Survivors of Childhood Cancer: A Report From the Childhood Cancer Survivor Study. *J Clin Oncol*. 2023:JCO2202320.

44. Errahmani MY, Locquet M, Spoor D, et al. Association between cardiac radiation exposure and the risk of arrhythmia in breast cancer patients treated with radiotherapy: A case-control study. *Front Oncol*. 2022;12:892882.

45. van Velzen SGM, Gal R, Teske AJ, et al. AI-Based Radiation Dose Quantification for Estimation of Heart Disease Risk in Breast Cancer Survivors After Radiation Therapy. *Int J Radiat Oncol Biol Phys*. 2022;112:621–632.

46. Zureick AH, Grzywacz VP, Almahariq MF, et al. Dose to the Left Anterior Descending Artery Correlates With Cardiac Events After Irradiation for Breast Cancer. *Int J Radiat Oncol Biol Phys*. 2022;114:130–139.

47. Locquet M, Spoor D, Crijns A, et al. Subclinical left ventricular dysfunction detected by speckle-tracking echocardiography in breast cancer patients treated with radiation therapy: A six-month follow-up analysis (MEDIRAD EARLY-HEART study). *Front Oncol*. 2022;12:883679.

48. Wang X, Palaskas NL, Hobbs BP, et al. The Impact of Radiation Dose to Heart Substructures on Major Coronary Events and Patient Survival after Chemoradiation Therapy for Esophageal Cancer. *Cancers* . 2022;14.

49. Nabialek-Trojanowska I, Sinacki M, Jankowska H, Lewicka-Potocka Z, Dziadziuszko R, Lewicka E. The influence of radiotherapy on the function of the left and right ventricles in relation to the radiation dose administered to the left anterior descending coronary artery-from a cardiologist’s Point of View. *Cancers* . 2022;14:2420.

50. Cho S-G, Kim Y-H, Park H, et al. Prediction of cardiac events following concurrent chemoradiation therapy for non-small-cell lung cancer using FDG PET. *Ann Nucl Med*. 2022;36:439–449.

51. Kim KH, Oh J, Yang G, et al. Association of sinoatrial node radiation dose with atrial fibrillation and mortality in patients with lung cancer. *JAMA Oncol*. 2022;8:1624–1634.

52. Tjong MC, Bitterman DS, Brantley K, et al. Major adverse cardiac event risk prediction model incorporating baseline Cardiac disease, Hypertension, and Logarithmic Left anterior descending coronary artery radiation dose in lung cancer (CHyLL). *Radiother Oncol*. 2022;169:105–113.

53. Tagami T, Almahariq MF, Balanescu DV, et al. Usefulness of Coronary Computed Tomographic Angiography to Evaluate Coronary Artery Disease in Radiotherapy-Treated Breast Cancer Survivors. *Am J Cardiol*. 2021;143:14–20.

54. Trivedi SJ, Tang S, Byth K, et al. Segmental cardiac radiation dose determines magnitude of regional cardiac dysfunction. *J Am Heart Assoc*. 2021;10:e019476.

55. van den Bogaard VAB, Spoor DS, van der Schaaf A, et al. The Importance of Radiation Dose to the Atherosclerotic Plaque in the Left Anterior Descending Coronary Artery for Radiation-Induced Cardiac Toxicity of Breast Cancer Patients? *International Journal of Radiation Oncology*Biology*Physics*. 2021;110:1350–1359.

56. van Aken ESM, van der Laan HP, Bijl HP, et al. Risk of ischaemic cerebrovascular events in head and neck cancer patients is associated with carotid artery radiation dose. *Radiother Oncol*. 2021;157:182–187.

57. Atkins KM, Chaunzwa TL, Lamba N, et al. Association of Left Anterior Descending Coronary Artery Radiation Dose With Major Adverse Cardiac Events and Mortality in Patients With Non–Small Cell Lung Cancer. *JAMA Oncol*. 2021;7:206–219.

58. Atkins KM, Bitterman DS, Chaunzwa TL, et al. Mean Heart Dose Is an Inadequate Surrogate for Left Anterior Descending Coronary Artery Dose and the Risk of Major Adverse Cardiac Events in Lung Cancer Radiation Therapy. *Int J Radiat Oncol Biol Phys*. 2021;110:1473–1479.

59. Xu T, Meng QH, Gilchrist SC, et al. Assessment of prognostic value of high-sensitivity cardiac troponin T for early prediction of chemoradiation therapy-induced cardiotoxicity in patients with non-small cell lung cancer: A secondary analysis of a prospective randomized trial. *Int J Radiat Oncol Biol Phys*. 2021;111:907–916.

60. Pao T-H, Chang W-L, Chiang N-J, et al. Pericardial effusion after definitive concurrent chemotherapy and intensity modulated radiotherapy for esophageal cancer. *Radiat Oncol*. 2020;15:48.

61. Takeuchi Y, Murakami Y, Kameoka T, et al. Analysis of cardiac toxicity after definitive chemoradiotherapy for esophageal cancer using a biological dose-volume histogram. *J Radiat Res*. 2020;61:298–306.

62. Umezawa R, Kadoya N, Ota H, et al. Dose-dependent radiation-induced myocardial damage in esophageal cancer treated with chemoradiotherapy: A prospective cardiac magnetic resonance imaging study. *Adv Radiat Oncol*. 2020;5:1170–1178.

63. Jang B-S, Cha M-J, Kim HJ, et al. Heart substructural dosimetric parameters and risk of cardiac events after definitive chemoradiotherapy for stage III non-small cell lung cancer. *Radiother Oncol*. 2020;152:126–132.

64. Niedzielski JS, Wei X, Xu T, et al. Development and application of an elastic net logistic regression model to investigate the impact of cardiac substructure dose on radiation-induced pericardial effusion in patients with NSCLC. *Acta Oncol*. 2020;59:1193–1200.

65. Abouegylah M, Braunstein LZ, Alm El-Din MA, et al. Evaluation of radiation-induced cardiac toxicity in breast cancer patients treated with Trastuzumab-based chemotherapy. *Breast Cancer Res Treat*. 2019;174:179–185.

66. van den Bogaard VAB, van Luijk P, Hummel YM, et al. Cardiac function after radiation therapy for breast cancer. *Int J Radiat Oncol Biol Phys*. 2019;104:392–400.

67. Walker V, Lairez O, Fondard O, et al. Early detection of subclinical left ventricular dysfunction after breast cancer radiation therapy using speckle-tracking echocardiography: association between cardiac exposure and longitudinal strain reduction (BACCARAT study). *Radiat Oncol*. 2019;14:204.

68. Wennstig AK, Garmo H, Isacsson U, et al. The relationship between radiation doses to coronary arteries and location of coronary stenosis requiring intervention in breast cancer survivors. *Radiat Oncol*. 2019;14:40.

69. Trivedi SJ, Choudhary P, Lo Q, et al. Persistent reduction in global longitudinal strain in the longer term after radiation therapy in patients with breast cancer. *Radiother Oncol*. 2019;132:148–154.

70. Borkenhagen JF, Bergom C, Rapp CT, Klawikowski SJ, Rein LE, Gore EM. Dosimetric predictors of cardiotoxicity in thoracic radiotherapy for lung cancer. *Clin Lung Cancer*. 2019;20:435–441.

71. Hotca A, Thor M, Deasy JO, Rimner A. Dose to the cardio-pulmonary system and treatment-induced electrocardiogram abnormalities in locally advanced non-small cell lung cancer. *Clin Transl Radiat Oncol*. 2019;19:96–102.

72. Xue J, Han C, Jackson A, et al. Doses of radiation to the pericardium, instead of heart, are significant for survival in patients with non-small cell lung cancer. *Radiother Oncol*. 2019;133:213–219.

73. Milgrom SA, Varghese B, Gladish GW, et al. Coronary Artery Dose-Volume Parameters Predict Risk of Calcification After Radiation Therapy. *JACC Cardiovasc Imaging*. 2019;27:268–279.

74. Mansouri I, Allodji RS, Hill C, et al. The role of irradiated heart and left ventricular volumes in heart failure occurrence after childhood cancer. *Eur J Heart Fail*. 2019;21:509–518.

75. Jagsi R, Griffith KA, Moran JM, et al. A randomized comparison of radiation therapy techniques in the management of node-positive breast cancer: Primary outcomes analysis. *Int J Radiat Oncol Biol Phys*. 2018;101:1149–1158.

76. Reshko LB, Kalman NS, Hugo GD, Weiss E. Cardiac radiation dose distribution, cardiac events and mortality in early-stage lung cancer treated with stereotactic body radiation therapy (SBRT). *J Thorac Dis*. 2018;10:2346–2356.

77. Yegya-Raman N, Wang K, Kim S, et al. Dosimetric Predictors of Symptomatic Cardiac Events After Conventional-Dose Chemoradiation Therapy for Inoperable NSCLC. *J Thorac Oncol*. 2018;13:1508–1518.

78. van den Bogaard VAB, Ta BDP, van der Schaaf A, et al. Validation and Modification of a Prediction Model for Acute Cardiac Events in Patients With Breast Cancer Treated With Radiotherapy Based on Three-Dimensional Dose Distributions to Cardiac Substructures. *J Clin Oncol*. 2017;35:1171–1178.

79. Ogino I, Watanabe S, Sakamaki K, Ogino Y, Kunisaki C, Kimura K. Dosimetric predictors of radiation-induced pericardial effusion in esophageal cancer. *Strahlenther Onkol*. 2017;193:552–560.

80. Hahn E, Jiang H, Ng A, et al. Late Cardiac Toxicity After Mediastinal Radiation Therapy for Hodgkin Lymphoma: Contributions of Coronary Artery and Whole Heart Dose-Volume Variables to Risk Prediction. *Int J Radiat Oncol Biol Phys*. 2017;98:1116–1123.

81. Vivekanandan S, Landau DB, Counsell N, et al. The Impact of Cardiac Radiation Dosimetry on Survival After Radiation Therapy for Non-Small Cell Lung Cancer. *Int J Radiat Oncol Biol Phys*. 2017;99:51–60.

82. Wang K, Eblan MJ, Deal AM, et al. Cardiac Toxicity After Radiotherapy for Stage III Non-Small-Cell Lung Cancer: Pooled Analysis of Dose-Escalation Trials Delivering 70 to 90 Gy. *J Clin Oncol*. 2017;35:1387–1394.

83. Wang K, Pearlstein KA, Patchett ND, et al. Heart dosimetric analysis of three types of cardiac toxicity in patients treated on dose-escalation trials for Stage III non-small-cell lung cancer. *Radiother Oncol*. 2017;125:293–300.

84. Cao L, Cai G, Chang C, et al. Early cardiac toxicity following adjuvant radiotherapy of left-sided breast cancer with or without concurrent trastuzumab. *Oncotarget*. 2016;7:1042–1054.

85. Cao L, Cai G, Chang C, et al. Diastolic dysfunction occurs early in HER2-positive breast cancer patients treated concurrently with radiation therapy and trastuzumab. *Oncologist*. 2015;20:605–614.

86. Skyttä T, Tuohinen S, Boman E, Virtanen V, Raatikainen P, Kellokumpu-Lehtinen P-L. Troponin T-release associates with cardiac radiation doses during adjuvant left-sided breast cancer radiotherapy. *Radiat Oncol*. 2015;10:141.

87. Moignier A, Broggio D, Derreumaux S, et al. Coronary stenosis risk analysis following Hodgkin lymphoma radiotherapy: A study based on patient specific artery segments dose calculation. *Radiother Oncol*. 2015;117:467–472.

88. Cutter DJ, Schaapveld M, Darby SC, et al. Risk of valvular heart disease after treatment for Hodgkin lymphoma. *J Natl Cancer Inst*. 2015;107.

89. de Ville de Goyet M, Brichard B, Robert A, et al. Prospective cardiac MRI for the analysis of biventricular function in children undergoing cancer treatments. *Pediatric Blood & Cancer*. 2015;62:867–874.

90. Cao L, Hu WG, Kirova YM, et al. Potential impact of cardiac dose–volume on acute cardiac toxicity following concurrent trastuzumab and radiotherapy. *Cancer/Radiothérapie*. 2014;18:119–124.

91. Tamari K, Isohashi F, Akino Y, et al. Risk factors for pericardial effusion in patients with stage I esophageal cancer treated with chemoradiotherapy. *Anticancer Res*. 2014;34:7389–7393.

92. Girinsky T, M’Kacher R, Lessard N, et al. Prospective coronary heart disease screening in asymptomatic Hodgkin lymphoma patients using coronary computed tomography angiography: results and risk factor analysis. *Int J Radiat Oncol Biol Phys*. 2014;89:59–66.

93. Dorth JA, Patel PR, Broadwater G, Brizel DM. Incidence and risk factors of significant carotid artery stenosis in asymptomatic survivors of head and neck cancer after radiotherapy. *Head Neck*. 2014;36:215–219.

94. Cella L, Liuzzi R, Conson M, D’Avino V, Salvatore M, Pacelli R. Multivariate normal tissue complication probability modeling of heart valve dysfunction in Hodgkin lymphoma survivors. *Int J Radiat Oncol Biol Phys*. 2013;87:304–310.

95. Erven K, Jurcut R, Weltens C, et al. Acute radiation effects on cardiac function detected by strain rate imaging in breast cancer patients. *Int J Radiat Oncol Biol Phys*. 2011;79:1444–1451.

96. Cella L, Liuzzi R, Conson M, et al. Dosimetric predictors of asymptomatic heart valvular dysfunction following mediastinal irradiation for Hodgkin’s lymphoma. *Radiother Oncol*. 2011;101:316–321.

97. Schytte T, Hansen O, Stolberg-Rohr T, Brink C. Cardiac toxicity and radiation dose to the heart in definitive treated non-small cell lung cancer. *Acta Oncol*. 2010;49:1058–1060.

98. Gayed IW, Liu HH, Wei X, et al. Patterns of cardiac perfusion abnormalities after chemoradiotherapy in patients with lung cancer. *J Thorac Oncol*. 2009;4:179–184.

99. Wei X, Liu HH, Tucker SL, et al. Risk factors for pericardial effusion in inoperable esophageal cancer patients treated with definitive chemoradiation therapy. *Int J Radiat Oncol Biol Phys*. 2008;70:707–714.

100. Marks LB, Yu X, Prosnitz RG, et al. The incidence and functional consequences of RT-associated cardiac perfusion defects. *Int J Radiat Oncol Biol Phys*. 2005;63:214–223.
